# Storytelling as Innovative Method to Improve the Recognition of Teledentistry among Adults: A Randomized Controlled Trial

**DOI:** 10.1155/2023/8814905

**Published:** 2023-10-10

**Authors:** Khalid Aboalshamat, Fayyad Alsharif, Bader Alsanei, Alaa Aljohani, Saad Aljabri, Rayan Salawati, Afnan Nassar

**Affiliations:** ^1^Dental Public Health Division, Preventative Dentistry Department, College of Dental Medicine, Umm Al-Qura University, Makkah, Saudi Arabia; ^2^College of Dentistry, Umm Al-Qura University, Makkah, Saudi Arabia

## Abstract

**Objective:**

Storytelling is an educational approach that encourages learners to create imaginative conceptions and think creatively. The aim of this study was to assess the effects of storytelling on improving awareness about teledentistry among adults in Saudi Arabia.

**Materials and Methods:**

A single-blinded parallel group randomized controlled study with 88 adult participants from Saudi Arabia. Participants were randomized into an intervention group (IG) or a control group (CG). The IG received teledentistry information using a storytelling method, while the CG received a flyer containing the same information. The participants were questioned three times: P1 before the intervention, P2 immediately after the intervention, and P3 1 month later.

**Results:**

A total of 83 participants completed all study steps. No significant knowledge score differences between IG and CG at P1, P2, or P3 nor in mean differences across time points. However, significant increase from P1 to P2 (*p* < 0.001) and P1 to P3 (*p* < 0.001) via paired *t*-test; no change from P2 to P3 in IG (*p* = 0.99) or CG (*p* = 0.816). Storytelling was significantly more innovative and emotionally touching than conventional flyer. The study was registered with the number ISRCTN68587630.

**Conclusion:**

Storytelling was found to be a more innovative and emotionally impactful approach for promoting health compared to traditional flyers. Future studies should extend follow-up periods and explore diverse stories for external validation of this method.

## 1. Introduction

In 2003, the World Health Organization indicated that the focus of oral health education should be on behaviors for improving oral health or lowering the risk of oral diseases [1]. One approach that can be used to change such behaviors is storytelling [2]. Storytelling is a type of approach that encourages learners to create imaginative conceptions and think creatively [3]. Story-related events and concepts are arranged in a framework through tales, allowing for better comprehension and future recall of the knowledge imparted in the stories [4, 5]. Reading stories has become easier with phones and computers and they can be used for education, as well as entertainment [6]. Storytelling may lead to the storyteller and audience developing a sense of trust and comfort in a kind of relationship [7]. In some cultures, such as in Saudi Arabia, storytelling might be more relevant and culturally acceptable due to the heritage of storytelling found in the holy book of Muslim, Quran, as well as other holy books [8].

A number of interventional studies have used storytelling in the field of dentistry. Studies in the Philippines [8] and Malaysia [9] found storytelling to be effective in reducing children's dental anxiety, and storytelling was used to improve oral hygiene in Indonesia [10]. Other interventional studies among dental students found storytelling to be effective for improving recall of information, teamwork, and reflection, and for achieving a deep understanding [11]. In addition, storytelling was used in a dental anatomy class and found more satisfactory as a learning method when compared with the conventional direct teaching method [12]. One interesting interventional study among Native American and Indigenous Alaskan populations indicated that storytelling significantly increased knowledge and favorable beliefs regarding oral health, and the effects lasted 6 months after the intervention [13]. The authors emphasized that story length, content, and mode of delivery were important for proper engagement and relatability [13]. However, none of these studies used a rigorous study design, such as a randomized controlled trial. The effectiveness of such methods should be tested more in studies aimed at increasing awareness of various aspects of oral health to build a cohort of results.

In recent years, extensive technological innovations have been seen in dentistry, and a new term has strongly emerged in the research arena: teledentistry. Teledentistry encompasses the use of telecommunications, digital images, and digital devices for the exchange of clinical information and images, enabling dental consultations, treatment planning, analysis, and follow-up across geographic distances [14]. One study found teledentistry to be helpful in increasing access to dental care and dental education, although most dentists were unaware of that ability [15]. Similarly, a local Saudi Arabian study indicated that dental professionals were not sufficiently aware of teledentistry [16]. In fact, the awareness with teledentistry might be a vital point to utilize such technology in Saudi Arabia, as there is a major change in the healthcare infrastructure to be technology-friendly based in Saudi Arabia Vision 2030. Studies that aim to improve the awareness of teledentistry using vigorous interventional designs, such as randomized controlled trials, are lacking.

### 1.1. Aim

The aim of this study was to assess the effects of storytelling on improving awareness about teledentistry among adults in Saudi Arabia.

## 2. Materials and Methods

### 2.1. Study Design and Participants

**Figure 1:**
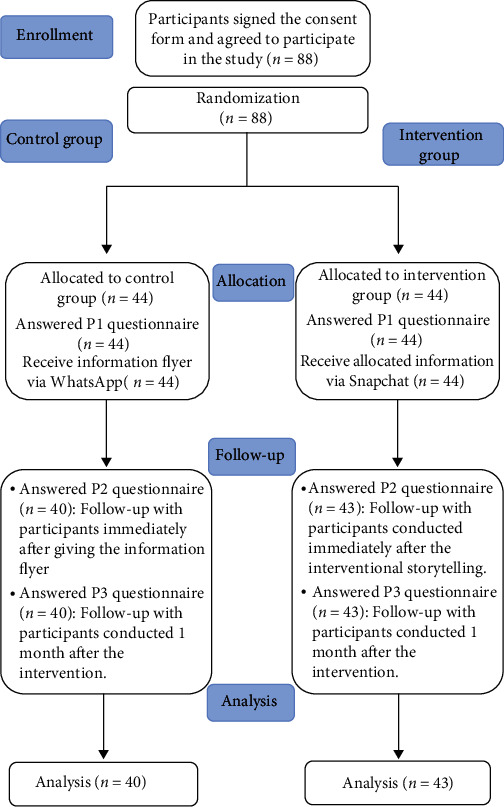
Participant flowchart.

This study was conducted using a single-blinded parallel group randomized controlled trial design. The intervention group (IG) received information about teledentistry by listening to a customized story created by the research team, while the control group (CG) received the same information about teledentistry through a traditional written flyer. The study's design is shown in Figure 1.

The study was written in alignment with the CONSORT statement checklist. The inclusion criteria were Arabic speakers who lived in Saudi Arabia and were 18 years old or older. Potential participants who did not agree to sign the consent form or who did not complete the three questionnaires were excluded from the study. Recruiting started at November 20, 2021. Participation in this study was voluntary, and participants were recruited via personal invitations and flyer advertisements distributed among dental patients and their relatives at Umm Al-Qura University Dental Hospital in Makkah, Saudi Arabia. The sample size for the study was calculated using the equation for randomized controlled trials with two independent samples, continuous outcomes, and a two-tailed hypothesis [17]:

(1) $$\begin{array}{c} n\left(\text{per group}\right)=2{\left(\frac{Z_{1-\alpha /2}+Z_{\beta -1}}{\text{ES}}\right)}^{2}, \end{array}$$

(2) $$\begin{array}{c} \text{ES}=\left(\frac{\text{Minimal clinical difference}}{\text{Standard devation}}\right). \end{array}$$

**Figure 2:**
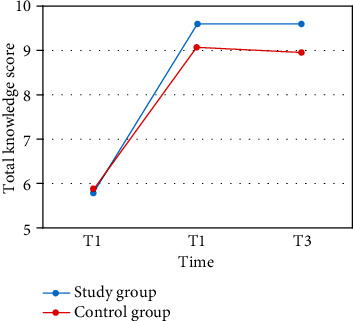
Trajectory of changes in total knowledge scores over time.

In the equation (Figure 2), ES means effect size. The alpha level was 5% (a = 0.05) and B = 0.1, so the constant Z(*β*−1) = 1.282 and constant *Z*(1−*α*/2) = 1.96 were used. A study power of 90% was selected, and the standard deviation (SD) of a previous study of knowledge about teledentistry was used (SD = 2.56) [16]. The minimal clinical difference was determined to be two points as assumption. The ES was calculated first to be 0.781 according to the above equation. The required minimum number of participants was 35 in each group, for a total of 70 participants. To compensate for an expected nonresponse rate, this number was multiplied by 1.5, resulting in 106 invitations needed for this study.

### 2.2. Setting

After the participants verbally agreed to participate in the study, the consent form was sent to them electronically as an online form. Communication with participants was through the WhatsApp application because it is widely accepted in Saudi Arabia and offers high levels of security [18] to ensure participant confidentiality. Participants were randomly assigned to a group using pieces of paper in a bowl with their ID number; a simple randomization procedure was conducted utilized prior prepared sealed envelopes with an equal allocation ratio, such that each participant was assigned randomly to an envelope, resulting in an equal probability of being allocated to one of the two groups. The sealed envelopes were opaque and consecutively numbered to ensure allocation concealment. To maintain blindness, the participants were told that the study was to assess different methods of improving knowledge about teledentistry. This made the participants unaware of which group was of interest to the research team. Also, the statistical analyst was blinded by recoding the IG and CG into different codes that remained hidden until the data analysis was completed.

The participants were evaluated three times: P1 before the intervention, P2 immediately after the intervention, and P3 4 weeks after the intervention for follow-up to evaluate their retention of the information. All identifiable information was removed after data collection was completed.

### 2.3. Intervention and Control Groups

After the participants in the IG agreed to the informed consent form of the study, they were asked to answer the P1 questionnaire in an online format. After completing the questionnaire, the IG participants received a link to a video where they could listen to the story created by the research team while the script appeared on screen. At the end of the story, another link directed the participants to the P2 questionnaire in an online format. The times required to answer the P1 and P2 questionnaires were recorded. If the time lapse between a participant's completion of P1 and P2 was less than 6 min, they were to be excluded from the study because it implied that the participant had not spent the minimum time required to listen to the entire story. However, no participants had a difference of 6 min or less. The P3 questionnaire was sent to IG participants for follow-up 1 month after completing P1 and P2.

The interventional story described a retired dentist and her journey using teledentistry in an emotional script. The story included 11 items encompassing information taken from a prior article [16] and designed to inform the listener about teledentistry, The story was 2,564 words in Arabic spanning four pages, and the story recording was approximately 12 min (6 min at 2x playback speed). The story script and link can be requested from the study authors. The story was tested through several phases to be validated in terms of understanding, grammar, syntax, and structure via small pilot studies with 12 participants.

The participants in the CG had the same protocol, except that they received an educational flyer (via WhatsApp) containing the 11 points of information about teledentistry rather than the story intervention.

### 2.4. Assessment

The questionnaire was a self-administered soft copy available through a link in Arabic. The questionnaire had three sections: demographic data, knowledge about teledentistry, and experiences and perceptions of the method of delivery. The distribution of these sections is shown in Table 1.

The demographic section contained seven questions collecting variables that included gender, education level, age, occupation, nationality, interest in reading stories, and previous knowledge about teledentistry in general. The knowledge about teledentistry section was composed of 11 questions to assess the participants' existing levels of knowledge regarding teledentistry. These questions were derived from a previous study [16], and the possible answers were yes, no, and I do not know. Each question had one correct answer, and correct answers received one point. The total teledentistry knowledge score was calculated by adding the points for correct answers. The section assessing the experiences and perceptions of the IG/CG was composed of 10 questions, with answers on a scale from 1 (strongly disagree) to 5 (strongly agree). These questions were derived from a previous study [19] with modifications. The questionnaire was validated by a round of pilot phases with 12 participants to validate understanding, grammar, syntax, and logical flow. The results of the 12 pilot tests were not included in the main study analysis.

### 2.5. Incentives and Ethical Considerations

All identifying information related to the participants was removed after data collection was completed. Participation in this study was voluntary. All participants who completed all three questionnaires were entered into a random prize drawing for one of six 50 Saudi Riyal (USD 13.33) bookstore vouchers as incentive to complete the study. Agreement by signing the study's informed consent form was mandatory before participants could complete the study's questionnaires. The study was approved by the institutional review board of Umm Al-Qura University, Saudi Arabia, number HAP0-,02-K-012-2021-11-811, on November 3, 2021. The study is registered at ISRCTN registry with the number ISRCTN68587630 (November 9, 2021) and can be accessed from https://doi.org/10.1186/ISRCTN68587630.

### 2.6. Data Analysis

The data were organized and sorted using Excel software (Microsoft Corp., Redmond, WA, USA) and analyzed using SPSS software version 27 (IBM Corp., Armonk, NY, USA). The data were analyzed using Fisher's exact test, repeated measures ANOVA, and paired *t*-test, and a *p*-value of 0.05 was used as the level of significance. The data are presented as mean, SD, frequency, and percentages.

## 3. Results

There were 106 participants invited to this study, and 88 agreed to participate via informed consent (response rate = 83.01%). After randomization, there were 44 participants in the IG and another 44 in the CG. There were total of five dropouts (7.35%) from the study, as shown in Figure 1. Only the data from the 83 participants were analyzed in this study. Missing values were zero because we used a mandatory electronic format questionnaire.

The 83 participants completed the questionnaires for P1, P2, and P3. The demographic data of the participants are provided in Table 2. The participants' mean (*m*) age was 24.20 years with an SD of 5.15. A chi-square test, Fisher's exact test, and *t*-test showed no significant differences between the IG and CG in gender, nationality, education, frequency of dental visits, sources of information about oral health, and interest in reading or listening to stories. However, the CG had a significantly higher percentage than the IG of those who knew about teledentistry before participating in this study.

Table 3 shows the descriptive statistics, including the mean and SD of the total knowledge scores, with a range of 0 (minimum score) to 11 (maximum score), for P1, P2, and P3. Table 3 also shows the same statistics for the differences in scores between P1, P2, and P3.

According to the *t*-test, there were no significant differences between the IG and CG in total knowledge scores at P1, P2, or P3. There were also no significant differences between the IG and CG in the score differences between P1, P2, and P3, as shown in Table 3. However, a paired *t*-test showed a significant increase from P1 to P2 (*p* < 0.001) and from P1 to P3 (*p* < 0.001) in the IG but no significant difference from P2 to P3 (*p*=0.99). This was exactly the same for the CG, with a significant increase from P1 to P2 (*p* < 0.001) and from P1 to P3 (*p* < 0.001) but no significant difference from P2 to P3 (*p*=0.816). These results are shown in Figure 2.

The participants' experiences of receiving the information via storytelling for the IG and flyer via WhatsApp for the CG are shown in Table 4. The responses were given on a rating scale from 1 (strongly disagree) to 5 (strongly agree). According to the *t*-test, the majority were not significantly different. However, participants rated storytelling significantly higher for “emotionally touching” and the innovation items.

## 4. Discussion

The aim of this study was to assess the effects of storytelling on improving awareness of teledentistry among adults in Saudi Arabia. Our findings demonstrated that storytelling led to an immediate improvement in teledentistry knowledge. This improvement was sustained even during the 1-month information retention follow-up. However, this was not significantly different from the conventional method used with the CG. Nevertheless, the participants found storytelling significantly more innovative and emotionally touching than receiving the information via a flyer.

Our results suggest that storytelling is a promising method for improving knowledge in the field of health. In fact, our results support other studies that investigated the effects of storytelling for managing dental anxiety in children, improving oral hygiene, improving oral health, and remembering information among different populations (children and adults) in several countries and cultures [8, 9, 11–13, 20]. However, our study also differs from others found in the literature. Most importantly, this is the first study, to the best of our knowledge, to compare a storytelling method with a CG using a blinded randomized controlled trial study design. Our results found that storytelling was not statistically different from a conventional method at improving knowledge, which could cast doubt on the findings from previous studies, and raise the question of whether storytelling is a better choice for boosting knowledge about health issues.

To tackle this dilemma, some points must be raised. First, there is no single story that can be used in every health-care intervention. In other words, each intervention necessitates the crafting of a new story according to the topic, which requires additional creative skills [21] and effort. This might increase the burden of making such interventions for future studies. Our current data do not provide adequate information to determine whether this burden is worth the effort. It is suggested that we conduct a future cross-over randomized controlled trial study investigating patient interest in a story versus the conventional method of distributing educational information.

Second, the story's effectiveness may be influenced by several factors, including story length, content, mode of delivery [13], audience relevance [22], culture [13], country, and age group. These factors were not investigated in our study, and future studies might consider these factors during the intervention design.

Third, it should be noted that while storytelling was found to be more emotionally engaging, the IG participants did not have higher knowledge scores or better knowledge retention than the CG. Nonetheless, this emotional engagement might be beneficial in terms of acceptability, willingness to take action, or attitudes about teledentistry or other health-care topics of interest. In fact, one previous study found that an engaging story of a clinical case was more persuasive with opioid tapering among patients [22]. These aspects were not investigated in the present study, and we recommend including such areas in future studies because storytelling might have more important beneficial effects than solely recalling information.

Fourth, the previous study among Native American and Indigenous Alaskan populations found the positive results of storytelling lasted 6 months after the intervention [13]. This study did not have a long follow-up period for comparison with the control group. It is possible that it could be argued that storytelling may produce longer retention of information about teledentistry. However, such a claim cannot be verified without further follow-up. Therefore, future studies should have longer follow-up periods to weigh the efforts and benefits of such interventions. Despite these concerns, it is noteworthy that storytelling was an effective method for improving health-care knowledge with an acceptable retention time.

In this intervention, there was a focus on boosting knowledge and awareness about teledentistry, which is a hot emerging topic in health-care delivery and services [23], especially in Saudi Arabia [24]. In fact, after the COVID-19 pandemic, a major shift occurred in health-care services resulting in teledentistry no longer being considered a luxury but, rather, a necessity [25, 26]. Previous studies suggested that knowledge of teledentistry in Saudi Arabia was lacking, even among dental professionals [19]. That is why, our intervention aimed at improving knowledge and awareness about teledentistry among a larger segment of the Saudi population to boost the use of such services and increase access to dental care. This is especially important with the rapid changes occurring in Saudi Arabia, with telehealth and telemedicine making rapid steps forward in light of Saudi Arabia Vision 2030 [16, 27].

The demographic data of the participants showed acceptable ratio between male and female with young adults with majority having education between high school and bachelor. This might have impaction on the external validity of our results and more studies might be conducted for children and older participants to assess the impact of such method. In fact, there are many usages of teledentistry nowadays [28] with different approaches [29] that making the investigation of different aspects of teledentistry beneficial.

This might be one of few studies investigating storytelling as a method of improving knowledge of innovations in the health-care field using a rigorous scientific study design (randomized controlled trial). However, there are a number of challenges that need to be addressed in future studies. The follow-up period after the intervention was not long enough to accurately measure information retention. Also, the sample size and the convenience sampling method used reduce the external validity of applying the study on a larger scale in Saudi Arabia.

## 5. Conclusion

Storytelling is an effective, more innovative, and emotionally touching method of health-promoting intervention than conventional methods. However, it is recommended that future studies can be conducted using a cross-over randomized controlled trial design with longer follow-up periods and investigating participant engagement and persuasion. Furthermore, different story lengths, content, and modes of delivery should be investigated, along with the audience's relevance, culture, and age group as potential influential factors.

## Figures and Tables

**Table 1:** Contents of questionnaires at P1, P2, and P3.

Questionnaire time	Intervention group	Control group
P1 (before the intervention)	(i) Demographic information (ii) Knowledge about teledentistry	(i) Demographic information (ii) Knowledge about teledentistry

P2 (immediately after the intervention) at the same day	(i) Knowledge about teledentistry (ii) Experiences and perceptions of the storytelling intervention	(i) Knowledge about teledentistry (ii) Experiences and perceptions of the control group flyer

P3 (1 month after the intervention)	(i) Knowledge about teledentistry	(i) Knowledge about teledentistry

**Table 2:** Participant demographic data.

Variable		IG		CG	
		*n*	%	*n*	%
Gender	Male	19	44.2	24	60.0
	Female	24	55.8	16	40.0

Nationality	Saudi	43	100.0	38	95.0
	Non-Saudi	0	0.0	2	5.0

Education	Less than high school	0	0.0	0	0.0
	High school	12	27.9	6	15.0
	Bachelor	31	72.1	34	85.0
	Higher education	0	0.0	0	0.0

Do you frequently visit the dentist?	Yes	6	14.0	7	17.5
	No	9	20.9	5	12.5
	When needed	28	65.1	28	70.0

Sources used for information about oral health in general	Doctors	23	53.5	25	62.5
	Dentists	41	95.3	37	92.5
	Social media	23	53.5	28	70.0
	Internet	32	74.4	34	85.0
	TV	9	20.9	8	20.0
	Books	10	23.3	15	37.5
	Others	13	30.2	10	25.0

In general, do you have an interest in reading stories?	Yes	24	55.8	21	52.5
	No	19	44.2	19	47.5

In general, do you usually like to listen to stories?	Yes	37	86.0	35	87.5
	No	6	14.0	5	12.5

Did you know what teledentistry is prior to participating in this study? ^*∗*^	Yes	3	7.0	11	27.5
	No	40	93.0	29	72.5

*Note*.  ^*∗*^The CG had a significantly higher percentage than the IG of those who knew about teledentistry before participating in this study. IG, intervention group; CG, control group.

**Table 3:** The mean and standard deviation (SD) of total knowledge scores and differences for P1, P2, and P3.

Score	IG		CG		*p*-value
	*m*	SD	*m*	SD	
Total knowledge P1	5.81	3.47	5.9	3.38	0.909
Total knowledge P2	9.58	2.22	9.05	1.89	0.243
Total knowledge P3	9.58	2.04	8.95	2.22	0.182
Difference between P1 and P2	3.77	4.21	3.15	3.39	0.463
Difference between P1 and P3	3.77	3.47	3.05	3.3	0.337
Difference between P2 and P3	0	2.26	−0.1	2.7	0.856

*Note*. IG, intervention group; CG, control group.

**Table 4:** Participants' experiences receiving information via storytelling for the IG and by flyer via WhatsApp for the CG.

Statement	IG (storytelling)		CG (flyer via WhatsApp)		*p*-value
	*m*	SD	*m*	SD	
I am completely satisfied with the experience of receiving information from the story/flyer.	4.35	1.04	4.13	1.11	0.349
I found the storytelling/flyer emotionally touching.	4.33	0.99	2.65	1.19	<0.001
The story/flyer contained useful information.	4.26	1.2	4.2	1.16	0.830
The time it took to read/listen to the story/flyer was appropriate.	3.65	1.43	4.05	1.08	0.154
I found the story/flyer interesting.	4.16	1.09	3.95	1.04	0.365
The story/flyer was easy to understand.	4.56	0.83	4.27	0.99	0.162
The story/flyer was stimulating.	4.07	1.2	3.65	1.25	0.124
I will recommend this story/flyer to others.	3.93	1.32	3.73	1.18	0.456
I found the story/flyer suitable for my age.	4.53	0.91	4.13	1.09	0.068
Receiving information via storytelling/flyer was innovative.	4.58	0.96	3.4	1.3	<0.001

*Note*. IG, intervention group; CG, control group.

## Data Availability

All raw data that produce the results are available as supporting information file.

## Abbreviations

CG: Control group
RCT: Randomized controlled trial
SD: Standard deviation
SG: Study group
P1: Period 1 before the intervention
P2: Immediately after the intervention
P3: 1 month later.

## Supplementary Materials

Supplementary 1 Study ethical approval from the institutional review board of Umm Al-Qura University, Saudi Arabia, number HAP0-,02-K-012-2021-11-811.

Supplementary 2 Consort statement.

Supplementary 3 The study raw data.
