# Supplementary material for: Storytelling as Innovative Method to Improve the Recognition of Teledentistry among Adults: A Randomized Controlled Trial
Source: Int J Dent. 2023 Oct 10;2023:8814905. doi: 10.1155/2023/8814905 (PMC10581858; doi:10.1155/2023/8814905)
Supplement: Supplementary 1 — Study ethical approval from the institutional review board of Umm Al-Qura University, Saudi Arabia, number HAP0-,02-K-012-2021-11-811. [file 8814905.f1.pdf]

Approval No. (HAPO-02-K-012-2021-11-811)

FINAL APPROVAL FROM THE BIOMEDICAL RESEARCH ETHICS COMMITTEE

Principal Investigator: **Dr. Khalid T. Aboalshamat**	Faculty: **Dentistry**	Date: **3/11/2021**

Proposal Title (in English):

The Effect of Story Telling of Improving the Recognition of Teledentistry among Adults: Randomized Controlled Trail

The Biomedical Research Ethics Committee has evaluated and examined the above-mentioned research proposal and has found it to be in accordance with the specifications and conditions of the ethics of scientific research.

The Committee has accordingly granted the Principal Investigator final approval concerning the ethics of scientific research

Principle Investigator is permitted to:

- Initiate the implementation of scientific research procedures within faculty facilities and laboratories, in addition to the regional research centers and hospitals
- Publish in scientific journals

Responsibility of Principal Investigator:

- Must provide a written statement to the Vice presidency of post-graduate studies and scientific research regarding any changes in the research plan, the committee shall decide whether a new approval is needed or not.

Director of Biomedical Ethics Committee

Dr. Aiman M. Momenah
Faculty of Medicine
Umm Al-Qura University

وزارة التعليم - جامعة أم القرى - لجنة الأخلاقيات الحيوية
Ministry of Education - Umm Al-Qura University Biomedical Ethics

Registration No. in National committee of Bio Ethics: HAPO-02-K-012
