# Supplementary material for: Storytelling as Innovative Method to Improve the Recognition of Teledentistry among Adults: A Randomized Controlled Trial
Source: Int J Dent. 2023 Oct 10;2023:8814905. doi: 10.1155/2023/8814905 (PMC10581858; doi:10.1155/2023/8814905)
Supplement: Supplementary 3 — The study raw data. [file 8814905.f3.pdf]

NewSN	SN	Group	Gender	Age	Nationality	Education	Do you frec	Source: Do
2	81	Study grou	Male	23	Saudi	High schoo	No	No
5	18	Study grou	Male	24	Saudi	Bachelor	When neec	No
7	61	Study grou	Female	48	Saudi	High schoo	When neec	No
8	42	Study grou	Female	21	Saudi	Bachelor	Yes	Yes
9	19	Study grou	Male	21	Saudi	Bachelor	Yes	Yes
10	82	Study grou	Male	23	Saudi	Bachelor	When neec	No
11	63	Study grou	Female	21	Saudi	Bachelor	No	Yes
18	20	Study grou	Male	21	Saudi	Bachelor	When neec	Yes
19	46	Study grou	Female	19	Saudi	Bachelor	No	No
23	54	Study grou	Female	20	Saudi	Bachelor	No	No
26	41	Study grou	Female	22	Saudi	Bachelor	Yes	Yes
27	3	Study grou	Male	24	Saudi	High schoo	When neec	Yes
29	21	Study grou	Male	25	Saudi	Bachelor	When neec	Yes
31	39	Study grou	Female	21	Saudi	High schoo	When neec	No
33	22	Study grou	Female	21	Saudi	Bachelor	When neec	Yes
34	43	Study grou	Female	20	Saudi	High schoo	When neec	No
37	31	Study grou	Female	23	Saudi	Bachelor	When neec	Yes
39	78	Study grou	Female	26	Saudi	Bachelor	When neec	Yes
42	44	Study grou	Female	30	Saudi	Bachelor	When neec	Yes
45	10	Study grou	Male	28	Saudi	Bachelor	When neec	No
46	53	Study grou	Female	23	Saudi	Bachelor	When neec	No
47	56	Study grou	Female	30	Saudi	Bachelor	When neec	Yes
49	64	Study grou	Male	26	Saudi	Bachelor	Yes	No
53	5	Study grou	Male	24	Saudi	Bachelor	When neec	Yes
54	35	Study grou	Female	22	Saudi	High schoo	No	No
56	68	Study grou	Male	30	Saudi	Bachelor	No	No
57	30	Study grou	Female	19	Saudi	Bachelor	When neec	Yes
58	73	Study grou	Female	35	Saudi	Bachelor	No	Yes
59	79	Study grou	Female	25	Saudi	High schoo	When neec	Yes
61	36	Study grou	Female	21	Saudi	High schoo	Yes	Yes
63	84	Study grou	Female	20	Saudi	Bachelor	Yes	No
66	86	Study grou	Male	25	Saudi	Bachelor	When neec	No
69	48	Study grou	Female	20	Saudi	High schoo	No	Yes
70	77	Study grou	Female	31	Saudi	Bachelor	When neec	Yes
71	37	Study grou	Male	25	Saudi	Bachelor	When neec	No
73	33	Study grou	Female	20	Saudi	High schoo	When neec	No
77	62	Study grou	Male	24	Saudi	Bachelor	When neec	Yes
78	12	Study grou	Male	23	Saudi	Bachelor	When neec	No
79	51	Study grou	Female	20	Saudi	High schoo	When neec	No
81	69	Study grou	Male	28	Saudi	Bachelor	When neec	No
83	6	Study grou	Male	24	Saudi	Bachelor	When neec	Yes
86	8	Study grou	Male	21	Saudi	High schoo	When neec	Yes
87	15	Study grou	Male	24	Saudi	Bachelor	No	Yes
1	4	Control grc	Male	23	Saudi	Bachelor	When neec	Yes
3	1	Control grc	Male	24	Saudi	Bachelor	When neec	Yes
4	27	Control grc	Female	24	Saudi	Bachelor	Yes	Yes
6	60	Control grc	Female	52	Saudi	Bachelor	No	No
12	17	Control grc	Male	23	Saudi	High schoo	No	No
13	14	Control grc	Male	24	Saudi	Bachelor	No	No

14	34	Control grc	Female	23	Saudi	Bachelor	When neec	No
15	11	Control grc	Male	25	Non-Saudi	Bachelor	When neec	Yes
16	71	Control grc	Male	23	Saudi	Bachelor	When neec	No
17	83	Control grc	Female	20	Saudi	Bachelor	Yes	Yes
20	26	Control grc	Female	22	Saudi	Bachelor	Yes	Yes
21	13	Control grc	Male	22	Saudi	Bachelor	When neec	Yes
22	58	Control grc	Female	19	Saudi	Bachelor	When neec	Yes
24	75	Control grc	Male	29	Saudi	High schoo	When neec	Yes
25	52	Control grc	Female	21	Saudi	High schoo	When neec	Yes
28	2	Control grc	Male	24	Saudi	Bachelor	When neec	Yes
30	80	Control grc	Male	22	Saudi	Bachelor	No	Yes
32	45	Control grc	Male	25	Saudi	Bachelor	Yes	No
35	25	Control grc	Female	24	Saudi	Bachelor	When neec	No
38	59	Control grc	Male	25	Saudi	Bachelor	When neec	Yes
41	24	Control grc	Male	24	Saudi	Bachelor	When neec	No
43	9	Control grc	Male	24	Saudi	Bachelor	When neec	Yes
44	70	Control grc	Female	20	Saudi	Bachelor	When neec	No
48	40	Control grc	Female	25	Saudi	Bachelor	When neec	Yes
50	67	Control grc	Male	34	Saudi	Bachelor	When neec	No
51	65	Control grc	Male	22	Saudi	Bachelor	When neec	Yes
52	29	Control grc	Female	24	Saudi	Bachelor	When neec	Yes
55	16	Control grc	Male	25	Saudi	Bachelor	When neec	Yes
62	66	Control grc	Male	24	Saudi	Bachelor	When neec	No
64	28	Control grc	Female	23	Saudi	Bachelor	When neec	Yes
65	50	Control grc	Female	21	Saudi	Bachelor	When neec	No
68	76	Control grc	Male	21	Non-Saudi	High schoo	When neec	Yes
72	72	Control grc	Male	25	Saudi	High schoo	When neec	No
74	57	Control grc	Male	23	Saudi	Bachelor	When neec	Yes
75	47	Control grc	Female	21	Saudi	Bachelor	Yes	Yes
76	87	Control grc	Male	25	Saudi	Bachelor	When neec	Yes
80	85	Control grc	Male	26	Saudi	Bachelor	When neec	Yes
82	74	Control grc	Female	23	Saudi	High schoo	Yes	Yes
84	55	Control grc	Female	20	Saudi	Bachelor	No	No
85	7	Control grc	Male	24	Saudi	Bachelor	Yes	No

Source: De	Source: Soc	Source: Int	Source: TV	Source: Bo	Source: Otl	In general,	In general,	Do you knc
Yes	No	Yes	No	No	No	Yes	Yes	No
Yes	No	No	No	No	No	No	Yes	No
Yes	No	Yes	No	No	No	No	Yes	No
Yes	Yes	No	No	Yes	Yes	Yes	Yes	No
No	Yes	Yes	Yes	No	Yes	No	No	Yes
Yes	No	No	No	No	No	No	Yes	No
Yes	No	Yes	No	No	Yes	No	No	No
Yes	No	No	No	No	No	Yes	Yes	No
Yes	Yes	Yes	No	No	No	Yes	Yes	No
Yes	No	No	No	No	No	No	No	No
Yes	Yes	Yes	Yes	Yes	Yes	No	Yes	No
Yes	Yes	Yes	No	No	No	Yes	Yes	No
Yes	Yes	Yes	No	No	Yes	Yes	Yes	No
Yes	Yes	Yes	No	No	No	No	Yes	No
Yes	No	Yes	No	No	No	No	Yes	No
Yes	No	Yes	No	No	No	Yes	Yes	No
Yes	Yes	Yes	Yes	Yes	Yes	No	Yes	No
Yes	Yes	Yes	No	No	No	Yes	Yes	No
Yes	No	Yes	No	No	No	No	Yes	No
Yes	No	Yes	No	No	No	Yes	Yes	No
Yes	Yes	Yes	No	Yes	No	Yes	Yes	No
Yes	Yes	Yes	No	No	Yes	Yes	Yes	No
Yes	Yes	Yes	Yes	No	No	Yes	Yes	No
Yes	Yes	Yes	No	No	No	Yes	Yes	No
No	Yes	Yes	Yes	No	Yes	Yes	Yes	No
Yes	Yes	Yes	No	No	No	Yes	Yes	No
Yes	No	Yes	Yes	Yes	Yes	No	Yes	No
Yes	Yes	Yes	No	No	No	Yes	Yes	No
Yes	No	No	No	Yes	No	Yes	Yes	No
Yes	No	No	No	No	No	No	No	No
Yes	Yes	Yes	No	No	No	Yes	Yes	No
Yes	Yes	Yes	No	No	No	Yes	No	No
Yes	No	Yes	No	No	No	No	Yes	No
Yes	No	Yes	No	No	Yes	Yes	Yes	No
Yes	No	Yes	No	No	No	No	Yes	Yes
Yes	Yes	Yes	Yes	Yes	No	No	Yes	Yes
Yes	No	No	No	Yes	Yes	No	Yes	No
Yes	Yes	No	No	No	No	No	Yes	No
Yes	Yes	No	Yes	Yes	No	Yes	No	No
Yes	No	No	No	No	No	Yes	Yes	No
Yes	Yes	Yes	No	No	Yes	Yes	Yes	No
Yes	No	Yes	Yes	Yes	No	Yes	Yes	No
Yes	Yes	Yes	No	No	Yes	No	Yes	No
Yes	Yes	Yes	No	No	No	No	No	No
Yes	Yes	Yes	Yes	No	No	No	No	Yes
Yes	Yes	Yes	Yes	Yes	No	No	Yes	Yes
Yes	Yes	Yes	No	No	No	No	No	No
Yes	Yes	Yes	No	Yes	No	Yes	Yes	Yes
Yes	Yes	Yes	No	No	Yes	No	Yes	Yes

Yes	Yes	Yes	No	Yes	No	Yes	Yes	No
Yes	No	Yes	Yes	No	No	No	Yes	No
Yes	Yes	No	No	No	No	No	Yes	No
Yes	Yes	Yes	Yes	No	No	Yes	Yes	Yes
Yes	No	No	No	No	No	No	No	No
Yes	Yes	Yes	No	No	No	Yes	Yes	Yes
Yes	Yes	Yes	Yes	Yes	Yes	Yes	Yes	No
Yes	No	Yes	No	No	Yes	No	Yes	No
Yes	No	No	No	No	No	Yes	Yes	No
Yes	Yes	No	No	No	No	Yes	Yes	No
Yes	Yes	Yes	Yes	Yes	No	Yes	Yes	No
Yes	Yes	Yes	No	No	No	Yes	Yes	Yes
Yes	Yes	Yes	No	No	No	Yes	Yes	No
Yes	Yes	Yes	No	Yes	Yes	Yes	Yes	Yes
Yes	No	Yes	No	No	No	No	No	No
Yes	Yes	Yes	Yes	Yes	Yes	Yes	Yes	No
Yes	No	Yes	No	No	No	Yes	Yes	No
Yes	Yes	Yes	No	Yes	No	Yes	Yes	Yes
Yes	No	Yes	No	No	No	No	Yes	No
Yes	Yes	Yes	No	No	No	No	Yes	No
Yes	Yes	Yes	No	Yes	Yes	Yes	Yes	No
Yes	Yes	Yes	No	No	No	Yes	Yes	No
Yes	Yes	Yes	No	Yes	No	No	Yes	Yes
Yes	No	Yes	No	No	No	No	Yes	No
No	Yes	Yes	Yes	No	No	Yes	Yes	No
Yes	Yes	Yes	No	Yes	No	No	Yes	No
No	No	No	No	No	Yes	No	Yes	No
Yes	No	Yes	No	No	No	Yes	Yes	No
Yes	Yes	Yes	No	No	Yes	No	Yes	No
Yes	Yes	Yes	No	Yes	Yes	Yes	Yes	No
Yes	No	No	No	Yes	No	No	Yes	No
Yes	No	Yes	No	Yes	Yes	No	Yes	No
No	Yes	Yes	No	No	No	Yes	Yes	No
Yes	Yes	Yes	No	Yes	No	Yes	Yes	Yes

Monitor pa	Dental edu	Consult the	Early detec	Diagnosis a	Education a	Ease of pat	Can be use	Increasing t
Yes	Yes	Yes	Yes	Yes	No	Yes	Yes	Yes
I do not kn	I do not kn	Yes	Yes	Yes	Yes	Yes	Yes	Yes
I do not kn	I do not kn	Yes	Yes	I do not kn	I do not kn	Yes	I do not kn	Yes
Yes	No	Yes	Yes	Yes	No	Yes	Yes	No
I do not kn	Yes	Yes	No	Yes	No	Yes	Yes	I do not kn
Yes	I do not kn	I do not kn	I do not kn	I do not kn	I do not kn	I do not kn	I do not kn	I do not kn
I do not kn	I do not kn	I do not kn	I do not kn	I do not kn	I do not kn	I do not kn	I do not kn	I do not kn
No	Yes	Yes	Yes	Yes	No	No	Yes	No
I do not kn	I do not kn	I do not kn	I do not kn	I do not kn	I do not kn	I do not kn	I do not kn	I do not kn
I do not kn	I do not kn	I do not kn	I do not kn	I do not kn	I do not kn	I do not kn	I do not kn	I do not kn
Yes	Yes	Yes	Yes	Yes	Yes	Yes	Yes	Yes
I do not kn	I do not kn	I do not kn	No	No	I do not kn	I do not kn	Yes	Yes
Yes	I do not kn	Yes	Yes	Yes	I do not kn	Yes	Yes	Yes
Yes	I do not kn	I do not kn	Yes	I do not kn	Yes	Yes	Yes	I do not kn
Yes	I do not kn	Yes	Yes	Yes	No	Yes	Yes	I do not kn
Yes	No	No	Yes	Yes	I do not kn	I do not kn	Yes	Yes
Yes	No	Yes	Yes	Yes	Yes	Yes	Yes	Yes
I do not kn	I do not kn	Yes	Yes	Yes	I do not kn	Yes	I do not kn	I do not kn
Yes	Yes	Yes	Yes	Yes	Yes	No	I do not kn	Yes
I do not kn	I do not kn	I do not kn	I do not kn	I do not kn	I do not kn	I do not kn	I do not kn	I do not kn
I do not kn	I do not kn	I do not kn	I do not kn	I do not kn	Yes	Yes	I do not kn	I do not kn
I do not kn	I do not kn	I do not kn	I do not kn	I do not kn	I do not kn	I do not kn	Yes	Yes
I do not kn	I do not kn	I do not kn	I do not kn	I do not kn	I do not kn	I do not kn	I do not kn	I do not kn
Yes	I do not kn	I do not kn	Yes	I do not kn	I do not kn	Yes	I do not kn	I do not kn
Yes	Yes	Yes	Yes	Yes	I do not kn	I do not kn	I do not kn	I do not kn
No	I do not kn	I do not kn	I do not kn	No	Yes	Yes	I do not kn	Yes
Yes	Yes	Yes	Yes	Yes	No	Yes	Yes	Yes
Yes	I do not kn	Yes	Yes	Yes	I do not kn	Yes	Yes	Yes
Yes	No	Yes	I do not kn	No	I do not kn	Yes	Yes	I do not kn
Yes	I do not kn	Yes	Yes	No	I do not kn	Yes	Yes	Yes
I do not kn	Yes	I do not kn	Yes	I do not kn	I do not kn	Yes	Yes	I do not kn
Yes	No	Yes	I do not kn	Yes	No	Yes	Yes	Yes
Yes	No	Yes	Yes	Yes	I do not kn	Yes	Yes	Yes
Yes	Yes	Yes	Yes	Yes	Yes	Yes	Yes	Yes
Yes	Yes	I do not kn	Yes	I do not kn	No	Yes	No	Yes
Yes	Yes	Yes	Yes	Yes	Yes	Yes	Yes	Yes
Yes	No	Yes	No	No	No	Yes	Yes	I do not kn
I do not kn	I do not kn	I do not kn	I do not kn	I do not kn	I do not kn	I do not kn	I do not kn	I do not kn
No	Yes	Yes	Yes	Yes	No	Yes	No	No
No	Yes	Yes	Yes	Yes	Yes	Yes	I do not kn	I do not kn
Yes	Yes	Yes	Yes	Yes	I do not kn	Yes	I do not kn	Yes
I do not kn	I do not kn	I do not kn	I do not kn	I do not kn	I do not kn	I do not kn	I do not kn	I do not kn
I do not kn	I do not kn	No	No	No	Yes	Yes	Yes	No
I do not kn	I do not kn	I do not kn	I do not kn	I do not kn	I do not kn	I do not kn	I do not kn	I do not kn
Yes	Yes	I do not kn	No	No	Yes	Yes	Yes	I do not kn
Yes	Yes	Yes	Yes	Yes	Yes	Yes	Yes	Yes
I do not kn	I do not kn	I do not kn	I do not kn	I do not kn	I do not kn	I do not kn	Yes	I do not kn
Yes	Yes	Yes	Yes	Yes	Yes	Yes	Yes	Yes
Yes	No	Yes	Yes	No	No	Yes	Yes	Yes

I do not kn	Yes	I do not kn	Yes	I do not kn	Yes	I do not kn	I do not kn	I do not kn
I do not kn	I do not kn	I do not kn	I do not kn	I do not kn	I do not kn	I do not kn	I do not kn	I do not kn
I do not kn	I do not kn	Yes	Yes	I do not kn	I do not kn	Yes	I do not kn	I do not kn
Yes	Yes	No	No	No	Yes	Yes	Yes	Yes
Yes	Yes	Yes	Yes	I do not kn	Yes	Yes	Yes	Yes
Yes	No	No	No	No	Yes	No	Yes	Yes
Yes	Yes	I do not kn	Yes	I do not kn	Yes	Yes	Yes	Yes
Yes	I do not kn	I do not kn	Yes	Yes	I do not kn	I do not kn	I do not kn	I do not kn
Yes	Yes	Yes	Yes	Yes	Yes	Yes	No	Yes
No	I do not kn	Yes	No	No	Yes	No	Yes	Yes
Yes	I do not kn	Yes	Yes	No	No	Yes	I do not kn	Yes
Yes	Yes	Yes	No	No	Yes	Yes	Yes	No
Yes	Yes	Yes	Yes	Yes	Yes	Yes	Yes	Yes
Yes	Yes	Yes	Yes	Yes	Yes	Yes	Yes	Yes
Yes	No	Yes	Yes	I do not kn	I do not kn	Yes	I do not kn	Yes
I do not kn	No	Yes	Yes	Yes	No	Yes	Yes	No
Yes	I do not kn	Yes	Yes	I do not kn	Yes	Yes	I do not kn	Yes
Yes	Yes	Yes	Yes	No	No	Yes	Yes	Yes
I do not kn	I do not kn	Yes	I do not kn	No	Yes	Yes	I do not kn	No
I do not kn	No	Yes	Yes	I do not kn	I do not kn	Yes	I do not kn	Yes
Yes	I do not kn	Yes	I do not kn	I do not kn	Yes	No	Yes	I do not kn
No	Yes	Yes	No	No	No	Yes	I do not kn	No
Yes	Yes	Yes	I do not kn	No	No	Yes	I do not kn	Yes
No	I do not kn	Yes	I do not kn	Yes	No	Yes	Yes	Yes
Yes	I do not kn	I do not kn	Yes	I do not kn	I do not kn	Yes	I do not kn	I do not kn
I do not kn	I do not kn	I do not kn	I do not kn	I do not kn	I do not kn	I do not kn	I do not kn	I do not kn
I do not kn	I do not kn	Yes	I do not kn	No	No	Yes	Yes	Yes
Yes	I do not kn	Yes	Yes	Yes	Yes	Yes	Yes	Yes
Yes	I do not kn	Yes	Yes	I do not kn	I do not kn	Yes	Yes	I do not kn
I do not kn	I do not kn	I do not kn	I do not kn	I do not kn	I do not kn	I do not kn	I do not kn	I do not kn
Yes	I do not kn	Yes	No	No	No	Yes	Yes	I do not kn
I do not kn	I do not kn	I do not kn	I do not kn	I do not kn	I do not kn	I do not kn	I do not kn	I do not kn
I do not kn	I do not kn	I do not kn	I do not kn	I do not kn	I do not kn	I do not kn	I do not kn	I do not kn
Yes	Yes	Yes	Yes	I do not kn	I do not kn	Yes	Yes	Yes

Increasing a	Increase co	Do you knc	Monitor pa	Dental edu	Consult the	Early detec	Diagnosis a	Education a
Yes	Yes	No	Yes	I do not kn	I do not kn	Yes	I do not kn	I do not kn
Yes	Yes	No	Yes	No	Yes	Yes	Yes	Yes
Yes	I do not kn	Yes	Yes	I do not kn	Yes	Yes	Yes	No
Yes	Yes	Yes	Yes	Yes	Yes	Yes	Yes	Yes
Yes	Yes	Yes	Yes	No	Yes	Yes	No	No
I do not kn	I do not kn	Yes	Yes	Yes	Yes	Yes	Yes	Yes
I do not kn	I do not kn	Yes	Yes	No	Yes	Yes	No	No
Yes	Yes	Yes	Yes	Yes	Yes	No	No	Yes
I do not kn	I do not kn	Yes	Yes	Yes	Yes	Yes	Yes	Yes
I do not kn	Yes	Yes	Yes	Yes	Yes	Yes	Yes	Yes
Yes	Yes	Yes	Yes	Yes	Yes	Yes	Yes	Yes
Yes	Yes	Yes	Yes	Yes	Yes	Yes	Yes	Yes
Yes	Yes	No	Yes	I do not kn	I do not kn	I do not kn	I do not kn	I do not kn
Yes	Yes	Yes	Yes	Yes	Yes	Yes	Yes	Yes
No	Yes	Yes	Yes	Yes	Yes	Yes	Yes	Yes
Yes	Yes	Yes	Yes	Yes	Yes	Yes	Yes	Yes
Yes	Yes	Yes	Yes	Yes	Yes	Yes	Yes	Yes
I do not kn	I do not kn	Yes	Yes	Yes	Yes	Yes	Yes	Yes
Yes	Yes	Yes	Yes	Yes	Yes	Yes	Yes	Yes
I do not kn	I do not kn	No	Yes	I do not kn	Yes	Yes	Yes	Yes
Yes	I do not kn	Yes	Yes	Yes	Yes	Yes	Yes	Yes
No	No	Yes	Yes	Yes	Yes	Yes	Yes	Yes
I do not kn	I do not kn	Yes	Yes	No	Yes	Yes	Yes	Yes
I do not kn	Yes	Yes	Yes	No	Yes	Yes	Yes	No
Yes	Yes	Yes	Yes	Yes	Yes	Yes	Yes	Yes
Yes	Yes	Yes	Yes	Yes	Yes	I do not kn	Yes	Yes
Yes	Yes	Yes	Yes	Yes	Yes	Yes	Yes	Yes
Yes	Yes	Yes	Yes	No	Yes	Yes	Yes	No
Yes	I do not kn	Yes	Yes	Yes	Yes	Yes	Yes	I do not kn
Yes	Yes	Yes	Yes	Yes	Yes	Yes	Yes	Yes
I do not kn	I do not kn	Yes	Yes	Yes	Yes	Yes	Yes	Yes
Yes	Yes	Yes	Yes	Yes	Yes	Yes	Yes	Yes
Yes	Yes	Yes	Yes	Yes	Yes	Yes	Yes	Yes
Yes	Yes	Yes	Yes	Yes	Yes	Yes	Yes	Yes
Yes	I do not kn	Yes	Yes	Yes	Yes	Yes	Yes	Yes
Yes	Yes	Yes	Yes	Yes	Yes	Yes	Yes	Yes
Yes	I do not kn	Yes	Yes	Yes	Yes	No	No	Yes
I do not kn	I do not kn	Yes	Yes	I do not kn	Yes	Yes	Yes	Yes
Yes	Yes	Yes	Yes	Yes	Yes	Yes	Yes	Yes
I do not kn	I do not kn	Yes	Yes	Yes	Yes	Yes	Yes	Yes
Yes	Yes	No	Yes	Yes	Yes	Yes	Yes	Yes
I do not kn	I do not kn	Yes	Yes	Yes	Yes	Yes	Yes	I do not kn
Yes	Yes	Yes	Yes	Yes	No	Yes	Yes	No
I do not kn	I do not kn	Yes	Yes	Yes	Yes	Yes	Yes	Yes
Yes	Yes	Yes	Yes	Yes	Yes	Yes	No	Yes
Yes	Yes	Yes	Yes	Yes	Yes	Yes	Yes	Yes
Yes	Yes	Yes	Yes	No	Yes	Yes	Yes	Yes
Yes	Yes	Yes	Yes	Yes	Yes	Yes	Yes	Yes
Yes	No	Yes	Yes	No	Yes	Yes	Yes	Yes

Yes	I do not kn	Yes	Yes	Yes	Yes	Yes	Yes	Yes
I do not kn	I do not kn	No	Yes	I do not kn	Yes	Yes	I do not kn	Yes
Yes	I do not kn	No	I do not kn	I do not kn	I do not kn	Yes	Yes	I do not kn
Yes	Yes	Yes	Yes	Yes	Yes	Yes	Yes	Yes
Yes	Yes	Yes	Yes	I do not kn	Yes	Yes	Yes	Yes
No	No	Yes	Yes	Yes	Yes	Yes	Yes	No
Yes	Yes	Yes	Yes	Yes	Yes	I do not kn	Yes	Yes
I do not kn	I do not kn	Yes	Yes	Yes	Yes	Yes	Yes	I do not kn
Yes	Yes	Yes	Yes	Yes	Yes	Yes	Yes	Yes
No	I do not kn	Yes	Yes	I do not kn	Yes	Yes	I do not kn	Yes
No	No	Yes	Yes	Yes	Yes	Yes	Yes	Yes
Yes	Yes	Yes	Yes	No	Yes	No	No	Yes
Yes	Yes	Yes	Yes	Yes	Yes	Yes	Yes	Yes
Yes	Yes	Yes	Yes	Yes	Yes	Yes	Yes	Yes
Yes	Yes	Yes	Yes	Yes	Yes	No	No	Yes
I do not kn	Yes	Yes	Yes	Yes	Yes	Yes	Yes	Yes
Yes	Yes	Yes	Yes	Yes	Yes	I do not kn	I do not kn	Yes
No	Yes	Yes	Yes	Yes	Yes	Yes	I do not kn	Yes
Yes	Yes	Yes	Yes	Yes	Yes	Yes	Yes	Yes
I do not kn	Yes	Yes	Yes	I do not kn	Yes	Yes	Yes	Yes
Yes	Yes	Yes	Yes	Yes	Yes	Yes	Yes	Yes
Yes	Yes	Yes	Yes	Yes	Yes	Yes	Yes	Yes
Yes	Yes	Yes	Yes	Yes	Yes	Yes	Yes	Yes
Yes	Yes	Yes	Yes	Yes	Yes	Yes	No	Yes
Yes	I do not kn	Yes	Yes	No	Yes	Yes	Yes	Yes
I do not kn	I do not kn	No	Yes	Yes	Yes	Yes	Yes	Yes
Yes	Yes	Yes	Yes	Yes	Yes	Yes	I do not kn	Yes
Yes	Yes	Yes	Yes	No	Yes	Yes	Yes	No
Yes	Yes	Yes	Yes	No	Yes	Yes	I do not kn	No
I do not kn	I do not kn	Yes	Yes	Yes	Yes	Yes	Yes	Yes
Yes	Yes	Yes	Yes	No	Yes	Yes	Yes	No
I do not kn	I do not kn	No	Yes	Yes	I do not kn	I do not kn	Yes	I do not kn
I do not kn	I do not kn	No	Yes	No	Yes	Yes	I do not kn	No
Yes	Yes	Yes	Yes	No	I do not kn	Yes	Yes	I do not kn

Ease of pat	Can be use	Increasing	Increasing	Increase co	I am compl	I found the	The ... cont	The time it
Yes	Yes	I do not kn	Yes	I do not kn	Agree	Agree	Strongly Ag	Strongly Ag
No	No	Yes	Yes	No	Disagree	Strongly di	Strongly di	Disagree
Yes	Yes	Yes	Yes	I do not kn	Disagree	Strongly Ag	Strongly Ag	Agree
Yes	Yes	Yes	Yes	Yes	Agree	Neutral	Strongly di	Disagree
Yes	Yes	Yes	Yes	Yes	Strongly Ag	Neutral	Neutral	Neutral
Yes	Yes	Yes	Yes	Yes	Strongly Ag	Strongly Ag	Strongly Ag	Strongly Ag
Yes	I do not kn	I do not kn	Yes	I do not kn	Strongly Ag	Agree	Agree	Strongly Ag
Yes	Yes	No	No	Yes	Strongly Ag	Agree	Strongly Ag	Neutral
Yes	Yes	Yes	Yes	Yes	Agree	Strongly Ag	Neutral	Strongly di
Yes	Yes	Yes	Yes	Yes	Strongly Ag	Strongly Ag	Strongly Ag	Strongly Ag
Yes	I do not kn	I do not kn	Yes	I do not kn	Agree	Disagree	Strongly Ag	Agree
Yes	Yes	Yes	Yes	Yes	Strongly Ag	Strongly Ag	Strongly Ag	Strongly Ag
I do not kn	I do not kn	I do not kn	I do not kn	I do not kn	Strongly Ag	Strongly Ag	Strongly Ag	Strongly Ag
Yes	Yes	Yes	Yes	Yes	Agree	Neutral	Strongly Ag	Neutral
Yes	Yes	Yes	Yes	Yes	Strongly Ag	Agree	Disagree	Strongly di
Yes	Yes	Yes	Yes	Yes	Strongly Ag	Strongly Ag	Strongly Ag	Strongly di
Yes	Yes	Yes	Yes	Yes	Strongly Ag	Strongly Ag	Strongly Ag	Strongly Ag
Yes	Yes	Yes	Yes	Yes	Strongly Ag	Strongly Ag	Strongly Ag	Strongly Ag
Yes	Yes	Yes	Yes	Yes	Strongly Ag	Strongly Ag	Strongly Ag	Strongly Ag
Yes	Yes	Yes	Yes	Yes	Neutral	Strongly Ag	Disagree	Strongly di
Yes	Yes	Yes	Yes	Yes	Strongly Ag	Strongly Ag	Strongly Ag	Agree
Yes	Yes	Yes	Yes	Yes	Strongly Ag	Strongly Ag	Strongly Ag	Strongly Ag
Yes	Yes	Yes	Yes	Yes	Strongly Ag	Strongly Ag	Strongly Ag	Strongly Ag
Yes	Yes	Yes	Yes	Yes	Strongly Ag	Strongly Ag	Agree	Neutral
Yes	Yes	Yes	Yes	Yes	Strongly Ag	Strongly Ag	Strongly Ag	Agree
Yes	Yes	Yes	Yes	Yes	Agree	Agree	Agree	Neutral
Yes	Yes	Yes	Yes	Yes	Neutral	Neutral	Neutral	Disagree
Yes	Yes	Yes	Yes	Yes	Strongly Ag	Strongly Ag	Strongly Ag	Strongly Ag
Yes	Yes	Yes	Yes	Yes	Strongly Ag	Agree	Strongly Ag	Strongly Ag
Yes	Yes	Yes	Yes	Yes	Agree	Strongly Ag	Strongly Ag	Neutral
Yes	Yes	Yes	Yes	Yes	Strongly Ag	Agree	Strongly Ag	Neutral
Yes	Yes	Yes	Yes	Yes	Strongly Ag	Strongly Ag	Agree	Strongly Ag
Yes	Yes	Yes	Yes	Yes	Disagree	Agree	Strongly Ag	Disagree
Yes	Yes	Yes	Yes	Yes	Strongly Ag	Strongly Ag	Strongly Ag	Agree
Yes	Yes	Yes	Yes	Yes	Strongly Ag	Strongly Ag	Strongly Ag	Agree
Yes	Yes	Yes	Yes	Yes	Neutral	Neutral	Neutral	Disagree
Yes	I do not kn	Yes	Yes	No	Agree	Strongly Ag	Neutral	Strongly Ag
Yes	Yes	No	Yes	Yes	Strongly Ag	Strongly Ag	Strongly Ag	Strongly Ag
Yes	Yes	Yes	Yes	Yes	Strongly Ag	Neutral	Agree	Agree
I do not kn	Yes	Yes	Yes	Yes	Strongly Ag	Strongly Ag	Strongly Ag	Strongly Ag
Yes	Yes	Yes	Yes	Yes	Strongly Ag	Strongly Ag	Strongly Ag	Strongly Ag
Yes	Yes	Yes	I do not kn	Yes	Strongly di	Neutral	Disagree	Strongly di
No	I do not kn	Yes	Yes	No	Agree	Strongly Ag	Strongly Ag	Neutral
No	Yes	No	Yes	No	Agree	Strongly di	Disagree	Neutral
Yes	Yes	I do not kn	Yes	Yes	Strongly di	Disagree	Disagree	Strongly di
Yes	Yes	Yes	Yes	Yes	Agree	Agree	Neutral	Agree
Yes	No	Yes	Yes	Yes	Neutral	Neutral	Strongly Ag	Neutral
Yes	Yes	Yes	Yes	No	Agree	Disagree	Neutral	Agree
Yes	Yes	Yes	Yes	I do not kn	Agree	Disagree	Strongly Ag	Strongly Ag

Yes	Yes	Yes	Yes	Yes	Agree	Disagree	Agree	Agree
Yes	Yes	Yes	Yes	I do not kn	Agree	Neutral	Strongly Ag	Agree
Yes	I do not kn	I do not kn	I do not kn	I do not kn	Agree	Disagree	Agree	Neutral
Yes	Yes	Yes	Yes	Yes	Strongly Ag	Strongly Ag	Neutral	Neutral
Yes	I do not kn	Yes	Yes	Yes	Strongly Ag	Neutral	Strongly Ag	Strongly Ag
Yes	Yes	Yes	Yes	Yes	Strongly Ag	Neutral	Agree	Agree
Yes	I do not kn	I do not kn	Yes	Yes	Agree	Neutral	Strongly Ag	Strongly Ag
Yes	Yes	Yes	Yes	Yes	Strongly Ag	Strongly dis	Strongly Ag	Strongly Ag
Yes	Yes	Yes	Yes	Yes	Strongly Ag	Strongly dis	Strongly Ag	Strongly Ag
Yes	Yes	No	Yes	Yes	Agree	Agree	Agree	Agree
Yes	Yes	Yes	Yes	Yes	Neutral	Agree	Neutral	Neutral
Yes	Yes	Yes	Yes	Yes	Strongly Ag	Neutral	Strongly Ag	Agree
Yes	Yes	Yes	Yes	Yes	Agree	Disagree	Strongly Ag	Strongly Ag
Yes	Yes	Yes	Yes	Yes	Strongly Ag	Strongly Ag	Strongly Ag	Strongly Ag
Yes	Yes	No	Yes	Yes	Strongly Ag	Neutral	Strongly Ag	Agree
Yes	Yes	Yes	Yes	Yes	Agree	Strongly dis	Agree	Agree
Yes	I do not kn	Yes	Yes	Yes	Agree	Disagree	Strongly Ag	Strongly Ag
Yes	No	Yes	Yes	I do not kn	Strongly Ag	Agree	Strongly Ag	Strongly Ag
Yes	Yes	Yes	Yes	Yes	Strongly Ag	Neutral	Strongly Ag	Agree
Yes	Yes	Yes	Yes	Yes	Neutral	Disagree	Agree	Agree
Yes	Yes	Yes	Yes	Yes	Neutral	Neutral	Neutral	Neutral
Yes	No	No	Yes	Yes	Strongly Ag	Disagree	Agree	Agree
Yes	I do not kn	I do not kn	Yes	Yes	Agree	Disagree	Strongly Ag	Strongly Ag
Yes	I do not kn	Yes	Yes	Yes	Agree	Agree	Strongly Ag	Strongly Ag
Yes	I do not kn	Yes	Yes	Yes	Strongly Ag	Disagree	Strongly Ag	Strongly Ag
Yes	Yes	Yes	Yes	Yes	Strongly Ag	Strongly Ag	Strongly Ag	Strongly Ag
Yes	Yes	Yes	Yes	Yes	Strongly Ag	Disagree	Strongly Ag	Strongly Ag
Yes	Yes	Yes	Yes	Yes	Strongly Ag	Strongly Ag	Strongly Ag	Strongly Ag
Yes	Yes	I do not kn	Yes	Yes	Agree	Neutral	Strongly Ag	Strongly Ag
Yes	Yes	Yes	Yes	Yes	Strongly Ag	Disagree	Strongly Ag	Strongly Ag
Yes	I do not kn	I do not kn	Yes	Yes	Strongly Ag	Strongly dis	Agree	Agree
Yes	I do not kn	I do not kn	I do not kn	I do not kn	Strongly dis	Strongly dis	Strongly dis	Strongly dis
Yes	I do not kn	I do not kn	Yes	Yes	Strongly Ag	Disagree	Strongly Ag	Neutral
Yes	Yes	Yes	Yes	Yes	Strongly dis	Disagree	Strongly dis	Disagree

I find the …	The ... was	The ... was	I will recon	I found the	Receiving i	In general,	Monitor pa	Dental edu
Strongly Ag	Strongly Ag	Strongly Ag	Strongly Ag	Strongly Ag	Strongly Ag	Yes	Yes	Yes
Disagree	Disagree	Strongly di	Neutral	Agree	Neutral	No	Yes	No
Agree	Strongly Ag	Strongly Ag	Agree	Strongly Ag	Strongly Ag	Yes	Yes	No
Neutral	Strongly Ag	Agree	Agree	Agree	Agree	Yes	Yes	Yes
Agree	Strongly Ag	Strongly Ag	Agree	Strongly Ag	Strongly Ag	Yes	Yes	Yes
Strongly Ag	Strongly Ag	Strongly Ag	Strongly Ag	Strongly Ag	Strongly Ag	No	Yes	Yes
Strongly Ag	Strongly Ag	Agree	Strongly Ag	Strongly Ag	Strongly Ag	No	Yes	No
Agree	Strongly Ag	Agree	Strongly Ag	Agree	Strongly Ag	Yes	Yes	I do not kn
Agree	Strongly Ag	Agree	Strongly di	Neutral	Strongly Ag	Yes	Yes	I do not kn
Strongly Ag	Strongly Ag	Agree	Strongly Ag	Strongly Ag	Strongly Ag	No	Yes	Yes
Agree	Agree	Agree	Neutral	Strongly Ag	Strongly Ag	No	Yes	Yes
Strongly Ag	Agree	Agree	Neutral	Strongly Ag	Disagree	Yes	Yes	Yes
Strongly Ag	Strongly Ag	Strongly Ag	Strongly Ag	Strongly Ag	Strongly Ag	Yes	Yes	Yes
Neutral	Strongly Ag	Strongly Ag	Neutral	Strongly Ag	Strongly Ag	Yes	Yes	Yes
Strongly di	Agree	Strongly di	Strongly di	Strongly di	Strongly Ag	No	Yes	Yes
Agree	Strongly Ag	Agree	Agree	Agree	Strongly Ag	Yes	Yes	I do not kn
Strongly Ag	Strongly Ag	Strongly Ag	Strongly Ag	Strongly Ag	Strongly Ag	Yes	Yes	Yes
Strongly Ag	Strongly Ag	Strongly Ag	Strongly Ag	Strongly Ag	Strongly Ag	Yes	Yes	Yes
Strongly Ag	Strongly Ag	Strongly Ag	Strongly Ag	Strongly Ag	Strongly Ag	Yes	Yes	Yes
Disagree	Agree	Strongly di	Strongly di	Strongly Ag	Strongly Ag	Yes	Yes	Yes
Strongly Ag	Strongly Ag	Neutral	Agree	Strongly Ag	Strongly Ag	Yes	Yes	Yes
Strongly Ag	Strongly Ag	Strongly Ag	Strongly Ag	Strongly Ag	Strongly Ag	Yes	Yes	Yes
Strongly Ag	Strongly Ag	Strongly Ag	Strongly Ag	Strongly Ag	Strongly Ag	Yes	Yes	Yes
Strongly Ag	Strongly Ag	Agree	Strongly Ag	Strongly Ag	Strongly Ag	Yes	Yes	I do not kn
Strongly Ag	Strongly Ag	Strongly Ag	Agree	Strongly Ag	Agree	Yes	Yes	Yes
Strongly Ag	Strongly Ag	Strongly Ag	Strongly Ag	Strongly Ag	Strongly Ag	Yes	Yes	Yes
Neutral	Neutral	Neutral	Disagree	Agree	Agree	Yes	Yes	Yes
Strongly Ag	Strongly Ag	Strongly Ag	Strongly Ag	Strongly Ag	Strongly Ag	Yes	Yes	No
Strongly Ag	Strongly Ag	Strongly Ag	Strongly Ag	Strongly Ag	Strongly Ag	Yes	Yes	Yes
Agree	Strongly Ag	Strongly Ag	Agree	Strongly Ag	Strongly Ag	No	Yes	Yes
Agree	Strongly Ag	Strongly Ag	Strongly Ag	Strongly Ag	Strongly Ag	Yes	Yes	Yes
Strongly Ag	Neutral	Agree	Strongly Ag	Strongly Ag	Strongly Ag	Yes	Yes	Yes
Agree	Strongly Ag	Strongly Ag	Agree	Strongly Ag	Neutral	Yes	Yes	I do not kn
Agree	Strongly Ag	Agree	Strongly Ag	Strongly Ag	Strongly Ag	Yes	Yes	Yes
Strongly Ag	Strongly Ag	Agree	Strongly Ag	Strongly Ag	Strongly Ag	Yes	Yes	Yes
Disagree	Strongly Ag	Neutral	Disagree	Disagree	Disagree	No	Yes	Yes
Agree	Agree	Neutral	Neutral	Strongly Ag	Strongly Ag	No	Yes	I do not kn
Strongly Ag	Agree	Strongly Ag	Neutral	Agree	Agree	Yes	Yes	I do not kn
Neutral	Strongly Ag	Agree	Neutral	Strongly Ag	Strongly Ag	Yes	Yes	Yes
Strongly Ag	Agree	Agree	Strongly Ag	Neutral	Strongly Ag	Yes	Yes	Yes
Strongly Ag	Strongly Ag	Strongly Ag	Strongly Ag	Strongly Ag	Strongly Ag	Yes	Yes	Yes
Disagree	Neutral	Strongly di	Strongly di	Neutral	Strongly di	Yes	No	I do not kn
Agree	Disagree	Neutral	Neutral	Agree	Strongly Ag	Yes	Yes	Yes
Disagree	Strongly Ag	Neutral	Disagree	Neutral	Disagree	No	Yes	Yes
Disagree	Neutral	Strongly di	Disagree	Disagree	Strongly di	No	Yes	Yes
Agree	Neutral	Agree	Agree	Agree	Agree	No	Yes	Yes
Strongly Ag	Strongly Ag	Agree	Strongly Ag	Strongly Ag	Strongly Ag	No	Yes	Yes
Agree	Agree	Agree	Agree	Agree	Agree	Yes	Yes	Yes
Agree	Agree	Neutral	Agree	Agree	Agree	No	Yes	No

Agree	Agree	Agree	Agree	Agree	Agree	Yes	Yes	Yes
Agree	Agree	Neutral	Disagree	Agree	Agree	No	Yes	I do not kn
Agree	Strongly Ag	Strongly Ag	Disagree	Disagree	Neutral	No	No	Yes
Agree	Agree	Neutral	Agree	Agree	Agree	No	Yes	Yes
Strongly Ag	Strongly Ag	Strongly Ag	Strongly Ag	Strongly Ag	Strongly Ag	Yes	Yes	Yes
Neutral	Agree	Neutral	Agree	Strongly Ag	Agree	Yes	Yes	Yes
Strongly Ag	Strongly Ag	Agree	Neutral	Strongly Ag	Strongly Ag	Yes	Yes	Yes
Strongly Ag	Strongly Ag	Agree	Strongly di	Strongly Ag	Strongly Ag	Yes	Yes	Yes
Strongly Ag	Strongly Ag	Strongly di	Strongly Ag	Strongly Ag	Disagree	Yes	Yes	Yes
Agree	Agree	Neutral	Agree	Agree	Strongly di	Yes	No	I do not kn
Agree	Agree	Agree	Neutral	Neutral	Disagree	Yes	No	I do not kn
Neutral	Strongly Ag	Agree	Agree	Agree	Agree	Yes	Yes	Yes
Strongly Ag	Strongly Ag	Strongly Ag	Strongly Ag	Strongly Ag	Strongly Ag	Yes	Yes	Yes
Strongly Ag	Strongly Ag	Strongly Ag	Strongly Ag	Strongly Ag	Strongly Ag	Yes	Yes	Yes
Agree	Agree	Agree	Agree	Strongly Ag	Neutral	No	Yes	No
Agree	Neutral	Strongly di	Neutral	Agree	Neutral	Yes	Yes	Yes
Agree	Strongly Ag	Agree	Strongly Ag	Agree	Strongly Ag	Yes	Yes	Yes
Strongly Ag	Strongly Ag	Strongly Ag	Strongly Ag	Strongly Ag	Disagree	Yes	Yes	Yes
Neutral	Strongly Ag	Neutral	Agree	Strongly Ag	Agree	Yes	Yes	Yes
Neutral	Neutral	Neutral	Agree	Neutral	Neutral	Yes	Yes	I do not kn
Disagree	Neutral	Neutral	Neutral	Neutral	Agree	Yes	Yes	Yes
Agree	Strongly Ag	Agree	Strongly Ag	Strongly Ag	Disagree	Yes	Yes	No
Agree	Strongly Ag	Agree	Agree	Neutral	Neutral	No	Yes	Yes
Strongly Ag	Strongly Ag	Strongly Ag	Strongly Ag	Strongly Ag	Neutral	No	Yes	I do not kn
Strongly Ag	Strongly Ag	Strongly Ag	Strongly Ag	Strongly Ag	Neutral	Yes	Yes	Yes
Strongly Ag	Strongly Ag	Strongly Ag	Strongly Ag	Strongly Ag	Strongly Ag	No	I do not kn	I do not kn
Agree	Strongly Ag	Agree	Neutral	Strongly Ag	Agree	Yes	Yes	Yes
Strongly Ag	Strongly Ag	Strongly Ag	Strongly Ag	Strongly Ag	Strongly Ag	Yes	Yes	No
Strongly Ag	Strongly Ag	Agree	Agree	Strongly Ag	Disagree	No	Yes	No
Agree	Neutral	Neutral	Neutral	Neutral	Strongly di	Yes	Yes	I do not kn
Agree	Agree	Strongly Ag	Agree	Strongly Ag	Agree	No	Yes	I do not kn
Strongly di	Strongly di	Strongly di	Strongly di	Strongly di	Strongly di	Yes	Yes	Yes
Agree	Strongly Ag	Strongly Ag	Neutral	Strongly Ag	Agree	Yes	No	Yes
Disagree	Disagree	Strongly di	Disagree	Disagree	Disagree	Yes	Yes	Yes

Consult the	Early detec	Diagnosis a	Education a	Ease of pat	Can be use	Increasing t	Increasing a	Increase co
Yes	Yes	I do not kn	Yes	Yes	Yes	Yes	Yes	Yes
No	Yes	Yes	Yes	No	Yes	Yes	Yes	No
Yes	Yes	Yes	I do not kn	Yes	I do not kn	Yes	Yes	I do not kn
I do not kn	Yes	Yes	I do not kn	Yes	Yes	Yes	Yes	Yes
Yes	Yes	No	No	Yes	Yes	Yes	Yes	Yes
Yes	Yes	Yes	Yes	Yes	Yes	Yes	Yes	Yes
Yes	Yes	No	No	Yes	I do not kn	I do not kn	Yes	No
Yes	Yes	Yes	No	Yes	Yes	No	Yes	I do not kn
Yes	Yes	Yes	Yes	Yes	Yes	Yes	Yes	Yes
Yes	Yes	Yes	Yes	Yes	Yes	Yes	Yes	Yes
Yes	Yes	Yes	Yes	Yes	Yes	Yes	Yes	Yes
Yes	Yes	Yes	Yes	Yes	Yes	Yes	Yes	Yes
Yes	Yes	Yes	Yes	Yes	Yes	Yes	Yes	Yes
Yes	Yes	Yes	Yes	Yes	Yes	Yes	Yes	Yes
Yes	Yes	Yes	Yes	Yes	Yes	Yes	Yes	Yes
Yes	Yes	Yes	Yes	Yes	Yes	Yes	I do not kn	Yes
Yes	Yes	Yes	Yes	Yes	Yes	Yes	Yes	Yes
Yes	Yes	Yes	Yes	Yes	Yes	Yes	Yes	Yes
Yes	Yes	I do not kn	Yes	Yes	Yes	Yes	Yes	Yes
Yes	Yes	Yes	Yes	Yes	Yes	Yes	Yes	Yes
Yes	Yes	Yes	Yes	Yes	Yes	Yes	Yes	Yes
Yes	Yes	Yes	Yes	Yes	Yes	Yes	Yes	Yes
Yes	Yes	Yes	Yes	Yes	Yes	Yes	Yes	Yes
Yes	Yes	Yes	Yes	Yes	Yes	Yes	Yes	Yes
Yes	Yes	Yes	Yes	Yes	Yes	Yes	Yes	Yes
Yes	Yes	No	Yes	Yes	Yes	No	Yes	Yes
Yes	Yes	Yes	Yes	Yes	Yes	Yes	Yes	Yes
Yes	Yes	Yes	No	Yes	Yes	Yes	Yes	Yes
Yes	Yes	Yes	Yes	Yes	Yes	Yes	Yes	Yes
Yes	Yes	Yes	Yes	Yes	Yes	Yes	Yes	Yes
Yes	Yes	Yes	Yes	Yes	Yes	Yes	Yes	I do not kn
Yes	Yes	Yes	Yes	Yes	Yes	Yes	Yes	Yes
Yes	Yes	Yes	Yes	Yes	Yes	Yes	Yes	Yes
Yes	Yes	Yes	Yes	Yes	Yes	Yes	Yes	Yes
Yes	Yes	Yes	No	Yes	I do not kn	Yes	Yes	Yes
Yes	Yes	Yes	Yes	Yes	Yes	Yes	Yes	Yes
Yes	Yes	Yes	Yes	Yes	I do not kn	I do not kn	Yes	I do not kn
Yes	Yes	I do not kn	I do not kn	Yes	Yes	I do not kn	Yes	Yes
Yes	Yes	Yes	Yes	Yes	Yes	Yes	Yes	Yes
Yes	Yes	Yes	Yes	No	I do not kn	I do not kn	Yes	Yes
Yes	Yes	Yes	Yes	Yes	Yes	Yes	Yes	Yes
No	Yes	No	I do not kn	Yes	I do not kn	I do not kn	I do not kn	No
Yes	Yes	Yes	I do not kn	No	I do not kn	No	Yes	No
Yes	I do not kn	No	Yes	Yes	I do not kn	No	Yes	Yes
Yes	Yes	No	Yes	Yes	I do not kn	Yes	No	Yes
Yes	Yes	Yes	Yes	Yes	Yes	Yes	Yes	Yes
Yes	I do not kn	Yes	Yes	Yes	Yes	Yes	Yes	Yes
Yes	Yes	Yes	Yes	Yes	Yes	Yes	Yes	Yes
Yes	Yes	Yes	Yes	Yes	Yes	Yes	Yes	Yes

Yes	Yes	No	Yes	No	Yes	Yes	Yes	Yes
Yes	Yes	I do not kn	Yes	Yes	Yes	Yes	Yes	I do not kn
Yes	Yes	I do not kn	No	No	No	Yes	Yes	No
No	No	Yes	No	Yes	Yes	Yes	Yes	Yes
I do not kn	Yes	I do not kn	Yes	Yes	Yes	Yes	Yes	Yes
Yes	Yes	Yes	No	Yes	Yes	Yes	Yes	Yes
Yes	Yes	I do not kn	Yes	Yes	I do not kn	I do not kn	Yes	Yes
Yes	Yes	Yes	Yes	Yes	Yes	Yes	Yes	Yes
Yes	Yes	Yes	Yes	Yes	Yes	Yes	Yes	Yes
Yes	Yes	Yes	Yes	Yes	Yes	I do not kn	Yes	Yes
I do not kn	No	Yes	Yes	No	I do not kn	Yes	Yes	No
Yes	Yes	Yes	Yes	Yes	Yes	Yes	Yes	Yes
Yes	Yes	Yes	Yes	Yes	Yes	Yes	Yes	Yes
Yes	Yes	Yes	Yes	Yes	Yes	Yes	Yes	Yes
Yes	Yes	No	Yes	Yes	Yes	Yes	Yes	Yes
Yes	Yes	Yes	Yes	Yes	Yes	Yes	Yes	Yes
Yes	I do not kn	No	Yes	Yes	Yes	Yes	Yes	Yes
Yes	Yes	Yes	No	Yes	Yes	I do not kn	No	Yes
Yes	Yes	Yes	Yes	Yes	No	Yes	Yes	Yes
Yes	Yes	Yes	I do not kn	Yes	Yes	Yes	Yes	Yes
Yes	Yes	Yes	Yes	Yes	Yes	Yes	Yes	Yes
Yes	Yes	Yes	No	Yes	No	No	Yes	Yes
Yes	No	No	Yes	Yes	No	Yes	Yes	Yes
Yes	Yes	Yes	No	Yes	Yes	Yes	Yes	Yes
Yes	Yes	Yes	Yes	Yes	Yes	Yes	Yes	Yes
I do not kn	I do not kn	I do not kn	I do not kn	I do not kn	I do not kn	I do not kn	I do not kn	I do not kn
Yes	Yes	Yes	Yes	Yes	Yes	Yes	Yes	Yes
Yes	Yes	Yes	No	Yes	Yes	Yes	Yes	Yes
Yes	Yes	I do not kn	No	Yes	Yes	Yes	Yes	Yes
Yes	Yes	Yes	I do not kn	Yes	Yes	Yes	Yes	Yes
Yes	Yes	Yes	Yes	Yes	I do not kn	I do not kn	Yes	Yes
Yes	Yes	Yes	Yes	Yes	Yes	Yes	Yes	Yes
Yes	Yes	No	Yes	Yes	Yes	Yes	Yes	Yes
Yes	Yes	Yes	No	Yes	Yes	Yes	Yes	Yes

Monitor pa	Dental edu	Consult the	Early detec	Diagnosis a	Education a	Ease of pat	Can be use	Increasing t
Correct	Correct	Correct	Correct	Correct	Wrong	Correct	Correct	Correct
Wrong	Wrong	Correct	Correct	Correct	Correct	Correct	Correct	Correct
Wrong	Wrong	Correct	Correct	Wrong	Wrong	Correct	Wrong	Correct
Correct	Wrong	Correct	Correct	Correct	Wrong	Correct	Correct	Wrong
Wrong	Correct	Correct	Wrong	Correct	Wrong	Correct	Correct	Wrong
Correct	Wrong	Wrong	Wrong	Wrong	Wrong	Wrong	Wrong	Wrong
Wrong	Wrong	Wrong	Wrong	Wrong	Wrong	Wrong	Wrong	Wrong
Wrong	Correct	Correct	Correct	Correct	Wrong	Wrong	Correct	Wrong
Wrong	Wrong	Wrong	Wrong	Wrong	Wrong	Wrong	Wrong	Wrong
Wrong	Wrong	Wrong	Wrong	Wrong	Wrong	Wrong	Wrong	Wrong
Correct	Correct	Correct	Correct	Correct	Correct	Correct	Correct	Correct
Wrong	Wrong	Wrong	Wrong	Wrong	Wrong	Wrong	Correct	Correct
Correct	Wrong	Correct	Correct	Correct	Wrong	Correct	Correct	Correct
Correct	Wrong	Wrong	Correct	Wrong	Correct	Correct	Correct	Wrong
Correct	Wrong	Correct	Correct	Correct	Wrong	Correct	Correct	Wrong
Correct	Wrong	Wrong	Correct	Correct	Wrong	Wrong	Correct	Correct
Correct	Wrong	Correct	Correct	Correct	Correct	Correct	Correct	Correct
Wrong	Wrong	Correct	Correct	Correct	Wrong	Correct	Wrong	Wrong
Correct	Correct	Correct	Correct	Correct	Correct	Wrong	Wrong	Correct
Wrong	Wrong	Wrong	Wrong	Wrong	Wrong	Wrong	Wrong	Wrong
Wrong	Wrong	Wrong	Wrong	Wrong	Correct	Correct	Wrong	Wrong
Wrong	Wrong	Wrong	Wrong	Wrong	Wrong	Wrong	Correct	Correct
Wrong	Wrong	Wrong	Wrong	Wrong	Wrong	Wrong	Wrong	Wrong
Correct	Wrong	Wrong	Correct	Wrong	Wrong	Correct	Wrong	Wrong
Correct	Correct	Correct	Correct	Correct	Wrong	Wrong	Wrong	Wrong
Wrong	Wrong	Wrong	Wrong	Wrong	Correct	Correct	Wrong	Correct
Correct	Correct	Correct	Correct	Correct	Wrong	Correct	Correct	Correct
Correct	Wrong	Correct	Correct	Correct	Wrong	Correct	Correct	Correct
Correct	Wrong	Correct	Wrong	Wrong	Wrong	Correct	Correct	Wrong
Correct	Wrong	Correct	Correct	Wrong	Wrong	Correct	Correct	Correct
Wrong	Correct	Wrong	Correct	Wrong	Wrong	Correct	Correct	Wrong
Correct	Wrong	Correct	Wrong	Correct	Wrong	Correct	Correct	Correct
Correct	Wrong	Correct	Correct	Correct	Wrong	Correct	Correct	Correct
Correct	Correct	Correct	Correct	Correct	Correct	Correct	Correct	Correct
Correct	Correct	Wrong	Correct	Wrong	Wrong	Correct	Wrong	Correct
Correct	Correct	Correct	Correct	Correct	Correct	Correct	Correct	Correct
Correct	Wrong	Correct	Wrong	Wrong	Wrong	Correct	Correct	Wrong
Wrong	Wrong	Wrong	Wrong	Wrong	Wrong	Wrong	Wrong	Wrong
Wrong	Correct	Correct	Correct	Correct	Wrong	Correct	Wrong	Wrong
Wrong	Correct	Correct	Correct	Correct	Correct	Correct	Wrong	Wrong
Correct	Correct	Correct	Correct	Correct	Wrong	Correct	Wrong	Correct
Wrong	Wrong	Wrong	Wrong	Wrong	Wrong	Wrong	Wrong	Wrong
Wrong	Wrong	Wrong	Wrong	Wrong	Correct	Correct	Correct	Wrong
Wrong	Wrong	Wrong	Wrong	Wrong	Wrong	Wrong	Wrong	Wrong
Correct	Correct	Wrong	Wrong	Wrong	Correct	Correct	Correct	Wrong
Correct	Correct	Correct	Correct	Correct	Correct	Correct	Correct	Correct
Wrong	Wrong	Wrong	Wrong	Wrong	Wrong	Wrong	Correct	Wrong
Correct	Correct	Correct	Correct	Correct	Correct	Correct	Correct	Correct
Correct	Wrong	Correct	Correct	Wrong	Wrong	Correct	Correct	Correct

Wrong	Correct	Wrong	Correct	Wrong	Correct	Wrong	Wrong	Wrong
Wrong	Wrong	Wrong	Wrong	Wrong	Wrong	Wrong	Wrong	Wrong
Wrong	Wrong	Correct	Correct	Wrong	Wrong	Correct	Wrong	Wrong
Correct	Correct	Wrong	Wrong	Wrong	Correct	Correct	Correct	Correct
Correct	Correct	Correct	Correct	Wrong	Correct	Correct	Correct	Correct
Correct	Wrong	Wrong	Wrong	Wrong	Correct	Wrong	Correct	Correct
Correct	Correct	Wrong	Correct	Wrong	Correct	Correct	Correct	Correct
Correct	Wrong	Wrong	Correct	Correct	Wrong	Wrong	Wrong	Wrong
Correct	Correct	Correct	Correct	Correct	Correct	Correct	Wrong	Correct
Wrong	Wrong	Correct	Wrong	Wrong	Correct	Wrong	Correct	Correct
Correct	Wrong	Correct	Correct	Wrong	Wrong	Correct	Wrong	Correct
Correct	Correct	Correct	Wrong	Wrong	Correct	Correct	Correct	Wrong
Correct	Correct	Correct	Correct	Correct	Correct	Correct	Correct	Correct
Correct	Correct	Correct	Correct	Correct	Correct	Correct	Correct	Correct
Correct	Wrong	Correct	Correct	Wrong	Wrong	Correct	Wrong	Correct
Wrong	Wrong	Correct	Correct	Correct	Wrong	Correct	Correct	Wrong
Correct	Wrong	Correct	Correct	Wrong	Correct	Correct	Wrong	Correct
Correct	Correct	Correct	Correct	Wrong	Wrong	Correct	Correct	Correct
Wrong	Wrong	Correct	Wrong	Wrong	Correct	Correct	Wrong	Wrong
Wrong	Wrong	Correct	Correct	Wrong	Wrong	Correct	Wrong	Correct
Correct	Wrong	Correct	Wrong	Wrong	Correct	Wrong	Correct	Wrong
Wrong	Correct	Correct	Wrong	Wrong	Wrong	Correct	Wrong	Wrong
Correct	Correct	Correct	Wrong	Wrong	Wrong	Correct	Wrong	Correct
Wrong	Wrong	Correct	Wrong	Correct	Wrong	Correct	Correct	Correct
Correct	Wrong	Wrong	Correct	Wrong	Wrong	Correct	Wrong	Wrong
Wrong	Wrong	Wrong	Wrong	Wrong	Wrong	Wrong	Wrong	Wrong
Wrong	Wrong	Correct	Wrong	Wrong	Wrong	Correct	Correct	Correct
Correct	Wrong	Correct	Correct	Correct	Correct	Correct	Correct	Correct
Correct	Wrong	Correct	Correct	Wrong	Wrong	Correct	Correct	Wrong
Wrong	Wrong	Wrong	Wrong	Wrong	Wrong	Wrong	Wrong	Wrong
Correct	Wrong	Correct	Wrong	Wrong	Wrong	Correct	Correct	Wrong
Wrong	Wrong	Wrong	Wrong	Wrong	Wrong	Wrong	Wrong	Wrong
Wrong	Wrong	Wrong	Wrong	Wrong	Wrong	Wrong	Wrong	Wrong
Correct	Correct	Correct	Correct	Wrong	Wrong	Correct	Correct	Correct

Increasing a	Increase cc	Total know	Monitor pa	Dental edu	Consult the	Early detec	Diagnosis a	Education a
Correct	Correct	10	Correct	Wrong	Wrong	Correct	Wrong	Wrong
Correct	Correct	9	Correct	Wrong	Correct	Correct	Correct	Correct
Correct	Wrong	5	Correct	Wrong	Correct	Correct	Correct	Wrong
Correct	Correct	8	Correct	Correct	Correct	Correct	Correct	Correct
Correct	Correct	7	Correct	Wrong	Correct	Correct	Wrong	Wrong
Wrong	Wrong	1	Correct	Correct	Correct	Correct	Correct	Correct
Wrong	Wrong	0	Correct	Wrong	Correct	Correct	Wrong	Wrong
Correct	Correct	7	Correct	Correct	Correct	Wrong	Wrong	Correct
Wrong	Wrong	0	Correct	Correct	Correct	Correct	Correct	Correct
Wrong	Correct	1	Correct	Correct	Correct	Correct	Correct	Correct
Correct	Correct	11	Correct	Correct	Correct	Correct	Correct	Correct
Correct	Correct	4	Correct	Correct	Correct	Correct	Correct	Correct
Correct	Correct	9	Correct	Wrong	Wrong	Wrong	Wrong	Wrong
Correct	Correct	7	Correct	Correct	Correct	Correct	Correct	Correct
Wrong	Correct	7	Correct	Correct	Correct	Correct	Correct	Correct
Correct	Correct	7	Correct	Correct	Correct	Correct	Correct	Correct
Correct	Correct	10	Correct	Correct	Correct	Correct	Correct	Correct
Wrong	Wrong	4	Correct	Correct	Correct	Correct	Correct	Correct
Correct	Correct	9	Correct	Correct	Correct	Correct	Correct	Correct
Wrong	Wrong	0	Correct	Wrong	Correct	Correct	Correct	Correct
Correct	Wrong	3	Correct	Correct	Correct	Correct	Correct	Correct
Wrong	Wrong	2	Correct	Correct	Correct	Correct	Correct	Correct
Wrong	Wrong	0	Correct	Wrong	Correct	Correct	Correct	Correct
Wrong	Correct	4	Correct	Wrong	Correct	Correct	Correct	Wrong
Correct	Correct	7	Correct	Correct	Correct	Correct	Correct	Correct
Correct	Correct	5	Correct	Correct	Correct	Wrong	Correct	Correct
Correct	Correct	10	Correct	Correct	Correct	Correct	Correct	Correct
Correct	Correct	9	Correct	Wrong	Correct	Correct	Correct	Wrong
Correct	Wrong	5	Correct	Correct	Correct	Correct	Correct	Wrong
Correct	Correct	8	Correct	Correct	Correct	Correct	Correct	Correct
Wrong	Wrong	4	Correct	Correct	Correct	Correct	Correct	Correct
Correct	Correct	8	Correct	Correct	Correct	Correct	Correct	Correct
Correct	Correct	9	Correct	Correct	Correct	Correct	Correct	Correct
Correct	Correct	11	Correct	Correct	Correct	Correct	Correct	Correct
Correct	Wrong	6	Correct	Correct	Correct	Correct	Correct	Correct
Correct	Correct	11	Correct	Correct	Correct	Correct	Correct	Correct
Correct	Wrong	5	Correct	Correct	Correct	Wrong	Wrong	Correct
Wrong	Wrong	0	Correct	Wrong	Correct	Correct	Correct	Correct
Correct	Correct	7	Correct	Correct	Correct	Correct	Correct	Correct
Wrong	Wrong	6	Correct	Correct	Correct	Correct	Correct	Correct
Correct	Correct	9	Correct	Correct	Correct	Correct	Correct	Correct
Wrong	Wrong	0	Correct	Correct	Correct	Correct	Correct	Wrong
Correct	Correct	5	Correct	Correct	Wrong	Correct	Correct	Wrong
Wrong	Wrong	0	Correct	Correct	Correct	Correct	Correct	Correct
Correct	Correct	7	Correct	Correct	Correct	Correct	Wrong	Correct
Correct	Correct	11	Correct	Correct	Correct	Correct	Correct	Correct
Correct	Correct	3	Correct	Wrong	Correct	Correct	Correct	Correct
Correct	Correct	11	Correct	Correct	Correct	Correct	Correct	Correct
Correct	Wrong	7	Correct	Wrong	Correct	Correct	Correct	Correct

Correct	Wrong	4	Correct	Correct	Correct	Correct	Correct	Correct
Wrong	Wrong	0	Correct	Wrong	Correct	Correct	Wrong	Correct
Correct	Wrong	4	Wrong	Wrong	Wrong	Correct	Correct	Wrong
Correct	Correct	8	Correct	Correct	Correct	Correct	Correct	Correct
Correct	Correct	10	Correct	Wrong	Correct	Correct	Correct	Correct
Wrong	Wrong	4	Correct	Correct	Correct	Correct	Correct	Wrong
Correct	Correct	9	Correct	Correct	Correct	Wrong	Correct	Correct
Wrong	Wrong	3	Correct	Correct	Correct	Correct	Correct	Wrong
Correct	Correct	10	Correct	Correct	Correct	Correct	Correct	Correct
Wrong	Wrong	4	Correct	Wrong	Correct	Correct	Wrong	Correct
Wrong	Wrong	5	Correct	Correct	Correct	Correct	Correct	Correct
Correct	Correct	8	Correct	Wrong	Correct	Wrong	Wrong	Correct
Correct	Correct	11	Correct	Correct	Correct	Correct	Correct	Correct
Correct	Correct	11	Correct	Correct	Correct	Correct	Correct	Correct
Correct	Correct	7	Correct	Correct	Correct	Wrong	Wrong	Correct
Wrong	Correct	6	Correct	Correct	Correct	Correct	Correct	Correct
Correct	Correct	8	Correct	Correct	Correct	Wrong	Wrong	Correct
Wrong	Correct	8	Correct	Correct	Correct	Correct	Wrong	Correct
Correct	Correct	5	Correct	Correct	Correct	Correct	Correct	Correct
Wrong	Correct	5	Correct	Wrong	Correct	Correct	Correct	Correct
Correct	Correct	6	Correct	Correct	Correct	Correct	Correct	Correct
Correct	Correct	5	Correct	Correct	Correct	Correct	Correct	Correct
Correct	Correct	7	Correct	Correct	Correct	Correct	Correct	Correct
Correct	Correct	7	Correct	Correct	Correct	Correct	Wrong	Correct
Correct	Wrong	4	Correct	Wrong	Correct	Correct	Correct	Correct
Wrong	Wrong	0	Correct	Correct	Correct	Correct	Correct	Correct
Correct	Correct	6	Correct	Correct	Correct	Correct	Wrong	Correct
Correct	Correct	10	Correct	Wrong	Correct	Correct	Correct	Wrong
Correct	Correct	7	Correct	Wrong	Correct	Correct	Wrong	Wrong
Wrong	Wrong	0	Correct	Correct	Correct	Correct	Correct	Correct
Correct	Correct	6	Correct	Wrong	Correct	Correct	Correct	Wrong
Wrong	Wrong	0	Correct	Correct	Wrong	Wrong	Correct	Wrong
Wrong	Wrong	0	Correct	Wrong	Correct	Correct	Wrong	Wrong
Correct	Correct	9	Correct	Wrong	Wrong	Correct	Correct	Wrong

Ease of pat	Can be use	Increasing	Increasing	Increase co	Total know	Monitor pa	Dental edu	Consult the
Correct	Correct	Wrong	Correct	Wrong	5	Correct	Correct	Correct
Wrong	Wrong	Correct	Correct	Wrong	7	Correct	Wrong	Wrong
Correct	Correct	Correct	Correct	Wrong	8	Correct	Wrong	Correct
Correct	Correct	Correct	Correct	Correct	11	Correct	Correct	Wrong
Correct	Correct	Correct	Correct	Correct	8	Correct	Correct	Correct
Correct	Correct	Correct	Correct	Correct	11	Correct	Correct	Correct
Correct	Wrong	Wrong	Correct	Wrong	5	Correct	Wrong	Correct
Correct	Correct	Wrong	Wrong	Correct	7	Correct	Wrong	Correct
Correct	Correct	Correct	Correct	Correct	11	Correct	Wrong	Correct
Correct	Correct	Correct	Correct	Correct	11	Correct	Correct	Correct
Correct	Wrong	Wrong	Correct	Wrong	8	Correct	Correct	Correct
Correct	Correct	Correct	Correct	Correct	11	Correct	Correct	Correct
Wrong	Wrong	Wrong	Wrong	Wrong	1	Correct	Correct	Correct
Correct	Correct	Correct	Correct	Correct	11	Correct	Correct	Correct
Correct	Correct	Correct	Correct	Correct	11	Correct	Correct	Correct
Correct	Correct	Correct	Correct	Correct	11	Correct	Wrong	Correct
Correct	Correct	Correct	Correct	Correct	11	Correct	Correct	Correct
Correct	Correct	Correct	Correct	Correct	11	Correct	Correct	Correct
Correct	Correct	Correct	Correct	Correct	11	Correct	Correct	Correct
Correct	Correct	Correct	Correct	Correct	10	Correct	Correct	Correct
Correct	Correct	Correct	Correct	Correct	11	Correct	Correct	Correct
Correct	Correct	Correct	Correct	Correct	11	Correct	Correct	Correct
Correct	Correct	Correct	Correct	Correct	10	Correct	Correct	Correct
Correct	Correct	Correct	Correct	Correct	9	Correct	Wrong	Correct
Correct	Correct	Correct	Correct	Correct	11	Correct	Correct	Correct
Correct	Correct	Correct	Correct	Correct	10	Correct	Correct	Correct
Correct	Correct	Correct	Correct	Correct	11	Correct	Correct	Correct
Correct	Correct	Correct	Correct	Correct	9	Correct	Wrong	Correct
Correct	Correct	Correct	Correct	Correct	10	Correct	Correct	Correct
Correct	Correct	Correct	Correct	Correct	11	Correct	Correct	Correct
Correct	Correct	Correct	Correct	Correct	11	Correct	Correct	Correct
Correct	Correct	Correct	Correct	Correct	11	Correct	Correct	Correct
Correct	Correct	Correct	Correct	Correct	11	Correct	Wrong	Correct
Correct	Correct	Correct	Correct	Correct	11	Correct	Correct	Correct
Correct	Correct	Correct	Correct	Correct	11	Correct	Correct	Correct
Correct	Correct	Correct	Correct	Correct	11	Correct	Correct	Correct
Correct	Wrong	Correct	Correct	Wrong	7	Correct	Wrong	Correct
Correct	Correct	Wrong	Correct	Correct	9	Correct	Wrong	Correct
Correct	Correct	Correct	Correct	Correct	11	Correct	Correct	Correct
Wrong	Correct	Correct	Correct	Correct	10	Correct	Correct	Correct
Correct	Correct	Correct	Correct	Correct	11	Correct	Correct	Correct
Correct	Correct	Correct	Wrong	Correct	9	Wrong	Wrong	Wrong
Wrong	Wrong	Correct	Correct	Wrong	6	Correct	Correct	Correct
Wrong	Correct	Wrong	Correct	Wrong	8	Correct	Correct	Correct
Correct	Correct	Wrong	Correct	Correct	9	Correct	Correct	Correct
Correct	Correct	Correct	Correct	Correct	11	Correct	Correct	Correct
Correct	Wrong	Correct	Correct	Correct	9	Correct	Correct	Correct
Correct	Correct	Correct	Correct	Wrong	10	Correct	Correct	Correct
Correct	Correct	Correct	Correct	Wrong	9	Correct	Wrong	Correct

Correct	Correct	Correct	Correct	Correct	11 Correct	Correct	Correct
Correct	Correct	Correct	Correct	Wrong	8 Correct	Wrong	Correct
Correct	Wrong	Wrong	Wrong	Wrong	3 Wrong	Correct	Correct
Correct	Correct	Correct	Correct	Correct	11 Correct	Correct	Wrong
Correct	Wrong	Correct	Correct	Correct	9 Correct	Correct	Wrong
Correct	Correct	Correct	Correct	Correct	10 Correct	Correct	Correct
Correct	Wrong	Wrong	Correct	Correct	8 Correct	Correct	Correct
Correct	Correct	Correct	Correct	Correct	10 Correct	Correct	Correct
Correct	Correct	Correct	Correct	Correct	11 Correct	Correct	Correct
Correct	Correct	Wrong	Correct	Correct	8 Wrong	Wrong	Correct
Correct	Correct	Correct	Correct	Correct	11 Wrong	Wrong	Wrong
Correct	Correct	Correct	Correct	Correct	8 Correct	Correct	Correct
Correct	Correct	Correct	Correct	Correct	11 Correct	Correct	Correct
Correct	Correct	Correct	Correct	Correct	11 Correct	Correct	Correct
Correct	Correct	Wrong	Correct	Correct	8 Correct	Wrong	Correct
Correct	Correct	Correct	Correct	Correct	11 Correct	Correct	Correct
Correct	Wrong	Correct	Correct	Correct	8 Correct	Correct	Correct
Correct	Wrong	Correct	Correct	Wrong	8 Correct	Correct	Correct
Correct	Correct	Correct	Correct	Correct	11 Correct	Correct	Correct
Correct	Correct	Correct	Correct	Correct	10 Correct	Wrong	Correct
Correct	Correct	Correct	Correct	Correct	11 Correct	Correct	Correct
Correct	Wrong	Wrong	Correct	Correct	9 Correct	Wrong	Correct
Correct	Wrong	Wrong	Correct	Correct	9 Correct	Correct	Correct
Correct	Wrong	Correct	Correct	Correct	9 Correct	Wrong	Correct
Correct	Wrong	Correct	Correct	Correct	9 Correct	Correct	Correct
Correct	Correct	Correct	Correct	Correct	11 Wrong	Wrong	Wrong
Correct	Correct	Correct	Correct	Correct	10 Correct	Correct	Correct
Correct	Correct	Correct	Correct	Correct	9 Correct	Wrong	Correct
Correct	Correct	Wrong	Correct	Correct	7 Correct	Wrong	Correct
Correct	Correct	Correct	Correct	Correct	11 Correct	Wrong	Correct
Correct	Wrong	Wrong	Correct	Correct	7 Correct	Wrong	Correct
Correct	Wrong	Wrong	Wrong	Wrong	4 Correct	Correct	Correct
Correct	Wrong	Wrong	Correct	Correct	6 Wrong	Correct	Correct
Correct	Correct	Correct	Correct	Correct	8 Correct	Correct	Correct

Early detec	Diagnosis a	Education a	Ease of pat	Can be use	Increasing t	Increasing a	Increase cc	Total know
Correct	Wrong	Correct	Correct	Correct	Correct	Correct	Correct	10
Correct	Correct	Correct	Wrong	Correct	Correct	Correct	Wrong	7
Correct	Correct	Wrong	Correct	Wrong	Correct	Correct	Wrong	7
Correct	Correct	Wrong	Correct	Correct	Correct	Correct	Correct	9
Correct	Wrong	Wrong	Correct	Correct	Correct	Correct	Correct	9
Correct	Correct	Correct	Correct	Correct	Correct	Correct	Correct	11
Correct	Wrong	Wrong	Correct	Wrong	Wrong	Correct	Wrong	5
Correct	Correct	Wrong	Correct	Correct	Wrong	Correct	Wrong	7
Correct	Correct	Correct	Correct	Correct	Correct	Correct	Correct	10
Correct	Correct	Correct	Correct	Correct	Correct	Correct	Correct	11
Correct	Correct	Correct	Correct	Correct	Correct	Correct	Correct	11
Correct	Correct	Correct	Correct	Correct	Correct	Correct	Correct	11
Correct	Correct	Correct	Correct	Correct	Correct	Correct	Correct	11
Correct	Correct	Correct	Correct	Correct	Correct	Correct	Correct	11
Correct	Correct	Correct	Correct	Correct	Correct	Correct	Correct	11
Correct	Correct	Correct	Correct	Correct	Correct	Wrong	Correct	9
Correct	Correct	Correct	Correct	Correct	Correct	Correct	Correct	11
Correct	Correct	Correct	Correct	Correct	Correct	Correct	Correct	11
Correct	Wrong	Correct	Correct	Correct	Correct	Correct	Correct	10
Correct	Correct	Correct	Correct	Correct	Correct	Correct	Correct	11
Correct	Correct	Correct	Correct	Correct	Correct	Correct	Correct	11
Correct	Correct	Correct	Correct	Correct	Correct	Correct	Correct	11
Correct	Correct	Correct	Correct	Correct	Correct	Correct	Correct	11
Correct	Correct	Correct	Correct	Correct	Correct	Correct	Correct	10
Correct	Correct	Correct	Correct	Correct	Correct	Correct	Correct	11
Correct	Wrong	Correct	Correct	Correct	Wrong	Correct	Correct	9
Correct	Correct	Correct	Correct	Correct	Correct	Correct	Correct	11
Correct	Correct	Wrong	Correct	Correct	Correct	Correct	Correct	9
Correct	Correct	Correct	Correct	Correct	Correct	Correct	Correct	11
Correct	Correct	Correct	Correct	Correct	Correct	Correct	Correct	11
Correct	Correct	Correct	Correct	Correct	Correct	Correct	Wrong	10
Correct	Correct	Correct	Correct	Correct	Correct	Correct	Correct	11
Correct	Correct	Correct	Correct	Correct	Correct	Correct	Correct	10
Correct	Correct	Correct	Correct	Correct	Correct	Correct	Correct	11
Correct	Correct	Wrong	Correct	Wrong	Correct	Correct	Correct	9
Correct	Correct	Correct	Correct	Correct	Correct	Correct	Correct	11
Correct	Correct	Correct	Correct	Wrong	Wrong	Correct	Wrong	7
Correct	Wrong	Wrong	Correct	Correct	Wrong	Correct	Correct	7
Correct	Correct	Correct	Correct	Correct	Correct	Correct	Correct	11
Correct	Correct	Correct	Wrong	Wrong	Wrong	Correct	Correct	8
Correct	Correct	Correct	Correct	Correct	Correct	Correct	Correct	11
Correct	Wrong	Wrong	Correct	Wrong	Wrong	Wrong	Wrong	2
Correct	Correct	Wrong	Wrong	Wrong	Wrong	Correct	Wrong	6
Wrong	Wrong	Correct	Correct	Wrong	Wrong	Correct	Correct	7
Correct	Wrong	Correct	Correct	Wrong	Correct	Wrong	Correct	8
Correct	Correct	Correct	Correct	Correct	Correct	Correct	Correct	11
Wrong	Correct	Correct	Correct	Correct	Correct	Correct	Correct	10
Correct	Correct	Correct	Correct	Correct	Correct	Correct	Correct	11
Correct	Correct	Correct	Correct	Correct	Correct	Correct	Correct	10

Correct	Wrong	Correct	Wrong	Correct	Correct	Correct	Correct	9
Correct	Wrong	Correct	Correct	Correct	Correct	Correct	Wrong	8
Correct	Wrong	Wrong	Wrong	Wrong	Correct	Correct	Wrong	5
Wrong	Correct	Wrong	Correct	Correct	Correct	Correct	Correct	8
Correct	Wrong	Correct	Correct	Correct	Correct	Correct	Correct	9
Correct	Correct	Wrong	Correct	Correct	Correct	Correct	Correct	10
Correct	Wrong	Correct	Correct	Wrong	Wrong	Correct	Correct	8
Correct	Correct	Correct	Correct	Correct	Correct	Correct	Correct	11
Correct	Correct	Correct	Correct	Correct	Correct	Correct	Correct	11
Correct	Correct	Correct	Correct	Correct	Wrong	Correct	Correct	8
Wrong	Correct	Correct	Wrong	Wrong	Correct	Correct	Wrong	4
Correct	Correct	Correct	Correct	Correct	Correct	Correct	Correct	11
Correct	Correct	Correct	Correct	Correct	Correct	Correct	Correct	11
Correct	Correct	Correct	Correct	Correct	Correct	Correct	Correct	11
Correct	Wrong	Correct	Correct	Correct	Correct	Correct	Correct	9
Correct	Correct	Correct	Correct	Correct	Correct	Correct	Correct	11
Wrong	Wrong	Correct	Correct	Correct	Correct	Correct	Correct	9
Correct	Correct	Wrong	Correct	Correct	Wrong	Wrong	Correct	8
Correct	Correct	Correct	Correct	Wrong	Correct	Correct	Correct	10
Correct	Correct	Wrong	Correct	Correct	Correct	Correct	Correct	9
Correct	Correct	Correct	Correct	Correct	Correct	Correct	Correct	11
Correct	Correct	Wrong	Correct	Wrong	Wrong	Correct	Correct	7
Wrong	Wrong	Correct	Correct	Wrong	Correct	Correct	Correct	8
Correct	Correct	Wrong	Correct	Correct	Correct	Correct	Correct	9
Correct	Correct	Correct	Correct	Correct	Correct	Correct	Correct	11
Wrong	Wrong	Wrong	Wrong	Wrong	Wrong	Wrong	Wrong	0
Correct	Correct	Correct	Correct	Correct	Correct	Correct	Correct	11
Correct	Correct	Wrong	Correct	Correct	Correct	Correct	Correct	9
Correct	Wrong	Wrong	Correct	Correct	Correct	Correct	Correct	8
Correct	Correct	Wrong	Correct	Correct	Correct	Correct	Correct	9
Correct	Correct	Correct	Correct	Wrong	Wrong	Correct	Correct	8
Correct	Correct	Correct	Correct	Correct	Correct	Correct	Correct	11
Correct	Wrong	Correct	Correct	Correct	Correct	Correct	Correct	9
Correct	Correct	Wrong	Correct	Correct	Correct	Correct	Correct	10

ledge T3

-5	0	5
-2	-2	0
3	2	-1
3	1	-2
1	2	1
10	10	0
5	5	0
0	0	0
11	10	-1
10	10	0
-3	0	3
7	7	0
-8	2	10
4	4	0
4	4	0
4	2	-2
1	1	0
7	7	0
2	1	-1
10	11	1
8	8	0
9	9	0
10	11	1
5	6	1
4	4	0
5	4	-1
1	1	0
0	0	0
5	6	1
3	3	0
7	6	-1
3	3	0
2	1	-1
0	0	0
5	3	-2
0	0	0
2	2	0
9	7	-2
4	4	0
4	2	-2
2	2	0
9	2	-7
1	1	0
8	7	-1
2	1	-1
0	0	0
6	7	1
-1	0	1
2	3	1

7	5	-2
8	8	0
-1	1	2
3	0	-3
-1	-1	0
6	6	0
-1	-1	0
7	8	1
1	1	0
4	4	0
6	-1	-7
0	3	3
0	0	0
0	0	0
1	2	1
5	5	0
0	1	1
0	0	0
6	5	-1
5	4	-1
5	5	0
4	2	-2
2	1	-1
2	2	0
5	7	2
11	0	-11
4	5	1
-1	-1	0
0	1	1
11	9	-2
1	2	1
4	11	7
6	9	3
-1	1	2
